# Comprehensive assembly and analysis of the transcriptome of maritime pine developing embryos

**DOI:** 10.1186/s12870-018-1564-2

**Published:** 2018-12-29

**Authors:** Andreia S. Rodrigues, José J. De Vega, Célia M. Miguel

**Affiliations:** 1 Instituto de Biologia Experimental e Tecnológica (iBET), Apartado 12, 2780-901 Oeiras, Portugal; 2 Instituto de Tecnologia Química e Biológica António Xavier, Universidade Nova de Lisboa (ITQB NOVA), Av. da República, 2780-157 Oeiras, Portugal; 3 Earlham Institute, Norwich Research Park, Norwich, NR4 7UZ UK; 4 Universidade de Lisboa, Faculdade de Ciências, BioISI - Biosystems & Integrative Sciences Institute, Campo Grande, 1749-016 Lisbon, Portugal

**Keywords:** Zygotic embryo, *Pinus pinaster*, Embryogenesis, RNA-seq, Developmental stages, Transcript profiling

## Abstract

**Background:**

There are clear differences in embryo development between angiosperm and gymnosperm species. Most of the current knowledge on gene expression and regulation during plant embryo development has derived from studies on angiosperms species, in particular from the model plant *Arabidopsis thaliana*. The few published studies on transcript profiling of conifer embryogenesis show the existence of many putative embryo-specific transcripts without an assigned function. In order to extend the knowledge on the transcriptomic expression during conifer embryogenesis, we sequenced the transcriptome of zygotic embryos for several developmental stages that cover most of *Pinus pinaster* (maritime pine) embryogenesis.

**Results:**

Total RNA samples collected from five zygotic embryo developmental stages were sequenced with Illumina technology. A de novo transcriptome was assembled as no genome sequence is yet published for *Pinus pinaster*. The transcriptome of reference for the period of zygotic embryogenesis in maritime pine contains 67,429 transcripts, which likely encode 58,527 proteins. The annotation shows a significant percentage, 31%, of predicted proteins exclusively present in pine embryogenesis. Functional categories and enrichment analysis of the differentially expressed transcripts evidenced carbohydrate transport and metabolism over-representation in early embryo stages, as highlighted by the identification of many putative glycoside hydrolases, possibly associated with cell wall modification, and carbohydrate transport transcripts. Moreover, the predominance of chromatin remodelling events was detected in early to middle embryogenesis, associated with an active synthesis of histones and their post-translational modifiers related to increased transcription, as well as silencing of transposons.

**Conclusions:**

Our results extend the understanding of gene expression and regulation during zygotic embryogenesis in conifers and are a valuable resource to support further improvements in somatic embryogenesis for vegetative propagation of conifer species. Specific transcripts associated with carbohydrate metabolism, monosaccharide transport and epigenetic regulation seem to play an important role in pine early embryogenesis and may be a source of reliable molecular markers for early embryogenesis.

**Electronic supplementary material:**

The online version of this article (10.1186/s12870-018-1564-2) contains supplementary material, which is available to authorized users.

## Background

In higher plants, embryogenesis starts with the zygote formation and comprehends the whole developmental process that leads to a full mature and dormant embryo, enclosed by the seed tissues [1]. Most of current knowledge about plant embryogenesis derived from studies on angiosperm species, in particular from the model organism *Arabidopsis thaliana* (reviewed in [2]). However, gymnosperm and angiosperm lineages are estimated to have driven apart over 300 million years ago [3] and their differences, in particular at the embryogenic phase, are well known (reviewed by [4]). Molecular studies of embryogenesis in gymnosperms, and especially in conifers, have gained interest in the last few years (reviewed in [5, 6]). This has been driven by a better understanding of how the characteristic differences in embryo development between angiosperms and gymnosperms are established at the molecular level, and their evolutionary implications. Moreover, further improvement of somatic embryogenesis, an attractive technology for large scale vegetative propagation of economically important conifers, is largely dependent on additional knowledge about the basic processes controlling embryo development.

Next-generation sequencing (NGS) technologies applied to mRNA discover and profiling (RNA-seq) have proved useful to study plant gene regulation, in particular for the non-model species still missing a genome of reference (reviewed by [7, 8]). Large RNA sequencing projects such as the 1KP project alone achieved the transcriptomic sequencing in over 1000 different plant species which represents a huge effort with high impact in phylogenetic and land plant evolution studies [http://www.onekp.com; [9]]. RNA-seq data have considerably advanced our knowledge about the regulation of plant stress responses [10], plant development [11–13], synthesis of commercially/biotechnologically relevant plant products [14] or even the evolution of specific genes [15]. The embryo mRNA transcriptomes of several plant species, including rice (*Oryza sativa*) [16], maize (*Zea mays*) [17, 18], canola (*Brassica napus*) [19] and radish (*Raphanus sativus* L.) [20], have been generated by RNA-seq technology. In conifers, the transcriptome of early developmental stages of Scots pine (*Pinus sylvestris*) [21] dominant embryo has been profiled using high-throughput sequencing. Additionally, other studies addressing gymnosperm embryogenesis using NGS (reviewed by [22]) include the reports by Yakovlev et al. (2014) on embryo transcriptome changes in *Picea abies* under different temperature conditions [23], on the transcriptomes of embryogenic and non-embryogenic tissues of *Picea balfouriana* [24] on transcriptome comparative analysis of early somatic embryo formation and seed development in *Araucaria angustifolia* [25], on a comprehensive transcriptome survey of several *Pinus lambertiana* tissue types including embryos [26], on somatic embryo transcriptome profiling in *Picea abies* and [27] on the identification of carbohydrate-mediated responses associated with *Araucaria angustifolia* embryo formation.

Previously, a time-course transcriptomic study in *Pinus pinaster* pointed out the relevance of epigenetic regulators and specific transcription factors during the development of the embryo [28]. In such study, a cross-species microarray hybridization approach was followed, limiting the identification of candidate transcripts to the set of array probes derived from *Pinus taeda* root and needle tissues. Building upon that study, we extend here the scope of the transcriptomic analysis by using a high-throughput sequencing approach, with its known benefits over microarrays (reviewed in [29]), including the capacity to retrieve novel and/or lowly expressed transcripts, or alternative splice variants that might have been missed by microarray analysis [12]. In this work, we generated a species-specific transcriptome of the developing embryo aiming to have a significantly extended catalogue of maritime pine transcripts expressed during embryogenesis, targeting those transcripts with higher differences in expression during embryo development. By following this approach, we have identified over-represented processes, namely carbohydrate metabolism and epigenetic regulation, in specific phases of embryo development as well as specific transcripts involved. This unique resource in maritime pine further contributes for deepening our knowledge of the transcriptional activity during embryogenesis in conifers.

## Results

### Comprehensive transcriptome assembly

We performed RNA-seq on five embryogenesis stages (Day0, Day5, Day11, Day15 and Day25) according to a previously reported staging system [30], which cover the whole developmental period of the zygotic embryo of *P. pinaster*, up to the maturation stage. To capture the transcriptome landscape of the embryo and major differences in gene expression throughout development Illumina short-reads technology was used to sequence the RNA-seq libraries. In the absence of a published reference genome for *P. pinaster*, a comprehensive transcriptome assembly approach ([31]; reviewed in [32]) was adopted, which combines a de novo assembly of the reads using Trinity (version 2.0.6) [33] and a guided-assembly by mapping both the reads and de novo assembled transcripts against *P. taeda* genome (version 1.01) [34]. A total of ca. 319 M read-pairs were obtained, with an average of 63.8 M read-pairs per sample and each sample contributing from 44.9 M (14.1%) to 75.4 M (23.6%) read-pairs. A 5.3% of the reads was removed by the filtering steps. The resulting ca. 302 M read-pairs were subsequently used for de novo transcriptome assembly and mapping to the *P. taeda* genome. After obtaining the comprehensive transcriptome assembly, a total of 183.4 M read-pairs mapped in the correct distance and orientation (*Properly* paired) to this reference for expression analysis, which represents 57.5% of the raw reads (Table 1).

**Table 1:** RNA-seq and mapping statistics of *P. pinaster* embryo developmental stages

Embryo developmental stage	Day0	Day5	Day11	Day15	Day25
Read length^a^	PE 50 bp	PE 50 bp	PE 50 bp	PE 100 bp	PE 100 bp
Raw pairs of reads	72,632,308	55,550,140	44,898,746	75,433,086	70,463,384
Clean pairs of reads	72,472,236	55,413,990	44,803,454	66,982,962	62,388,454
Pairs of reads mapping in correct distance and orientation on *P. taeda* genome (*Properly* paired) used for assembly	61,626,536	45,975,573	37,763,000	60,334,604	55,692,971
Pairs of reads properly mapping on *P. pinaster* comprehensive transcriptome used for expression	44,178,974	33,660,866	28,187,082	41,345,428	35,994,032

^a^PE = paired-end

The final reference transcriptome of maritime pine zygotic embryogenesis contains 67,429 transcripts (deposited at DDBJ/ENA/GenBank under the accession GGEX01000000, https://www.ebi.ac.uk/ena/data/view/GGEX01000000), varying in length from 148 bp to 12,752 bp and with a mean length of 999 bp (Table 2).

**Table 2:** Statistics of the assembled transcriptome from *P. pinaster* embryo development

	Reference transcriptome
Total assembled transcripts	67,429
ExN50^a^	1653 bp
Shorter assembled transcript length (transcript)	148 bp (Pp11025)
Longer assembled transcript length (transcript)	12,752 bp (Pp28188)
Mean length	999 bp
Median length	627 bp
Transcripts without N’s bases	65,852
Mean gap percentage per transcript length	0.2%
Predicted coding transcripts	58,527
Predicted non-coding transcripts	8896

^a^ExN50 = transcript length metric that considers the top most highly expressed transcripts and means that at least 50% of the assembled transcript nucleotides were found in transcripts that were at least this length

### Transcriptome annotation

All ORF possibilities were generated from the newly assembled transcriptome, and only one per transcript (the longest one in case of multiple possibilities) was translated to generate the final proteome containing 58,527 proteins (Additional file 1). The annotation of the assembled developing embryo transcriptome was done using two sources of information: best reciprocal hits (BRH) to the proteomes of *P. taeda*, *P. lambertiana* and *A. thaliana*, and homology to proteins in NCBI databases (Additional file 2). The function and gene ontology (GO) terms from annotated BRHs were assigned back to the *P. pinaster* protein, to annotate as result 14,211 *P. pinaster* proteins. Blast2GO (version 3.1) was used to annotate the transcripts, starting from the BLASTX alignments of the transcriptome to the NCBI non-redundant proteins database (E-value < 10^− 3^, Additional file 3). Nearly 30,000 sequences were annotated with at least one GO term, and around 7500 transcripts had a homologous in the NCBI database but could not clearly be associated to a GO term. There were 28,780, 26,585 and 24,241 transcripts with at least one F:GO, P:GO or C:GO term, respectively. There are 16,056 transcripts with at least one GO term from each of the three categories. Over 20,000 *P. pinaster* protein sequences did not align to any protein in the database (Additional file 4). The homologous proteins presented a mean similarity of 71%, while 54% of the BLAST hits had a sequence similarity over 70% (Additional file 5). The analysis of the highest scoring homologous sequence to each *P. pinaster* transcripts showed that *Picea sitchensis*, a close relative of *P. pinaster*, is the most represented species by far, with over 16,000 *BLAST Top-Hits*. The other homologous belonged to species from different plant taxonomic groups, including gymnosperms, angiosperms and mosses. Four other *Pinus* species were represented, namely *P. taeda*, *P. radiata*, *P. sylvestris* and *P. monticola* (Additional file 6). The comparison with EBI’s InterPro database (IPS) for protein sequences and functions revealed about 37,500 *P. pinaster* sequences with a IPS result; IPS analysis contributed GO terms to over 20,000 *P. pinaster* sequences (Additional file 7). Mapping results revealed that UniProtKB and TAIR were the two main source databases of GO terms associated to *P. pinaster* sequences (Additional file 8).

### Functional regulation during embryo development

**Fig. 1:**
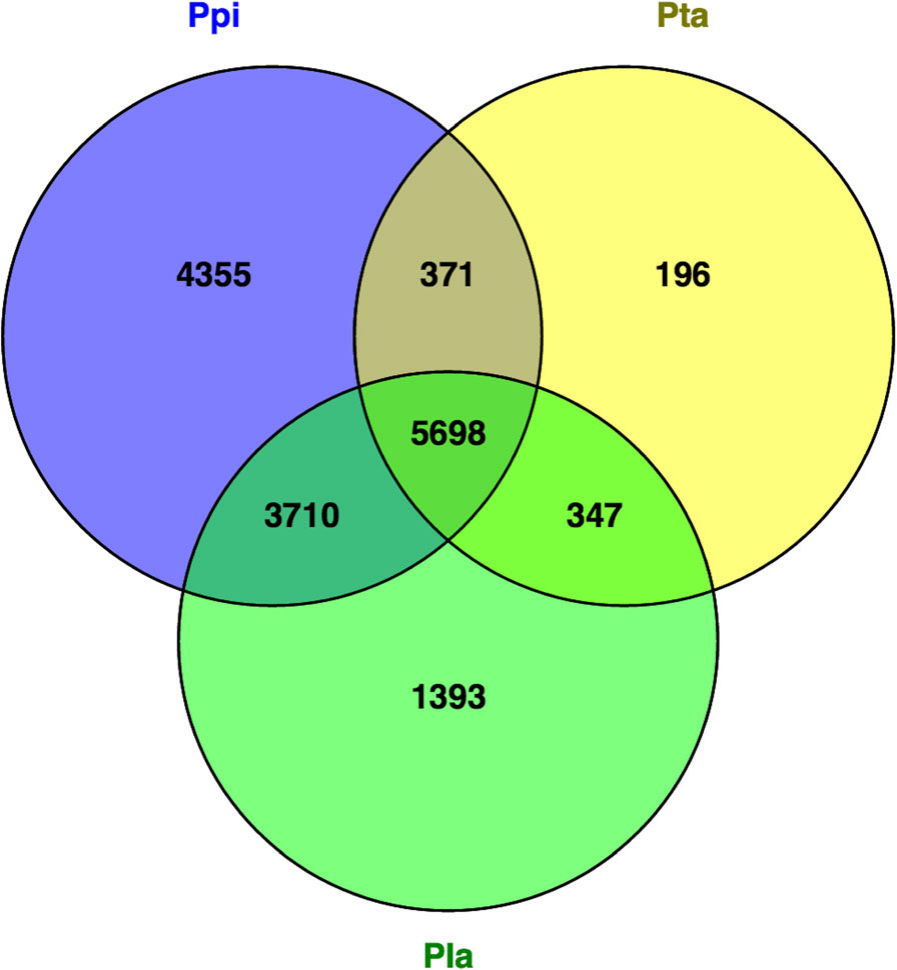
Venn diagram of the number of eggNOGs found with the predicted proteins of *P. pinaster* (Ppi) and two other relative conifer species, *P. taeda* (Pta) and *P. lambertiana* (Pla). Proteins were first annotated with the eggNOG numbers of their best homolog and then those sharing the same eggNOG number were concatenated. The numbers in the intersections represent the eggNOGs these conifers have in common

The proteins from *P. pinaster, P. taeda and P. lambertiana* were clustered together according to the eggNOG group of their respective best orthologous sequenced in EMBL’s eggNOG database of functionally annotated proteins (Additional file 9). When comparing the number of groups in the three species, *P. pinaster* had the highest number of exclusive groups (4355). Still, most of the groups, in a total of 5698, had proteins from the three species (Fig. 1). The groups with more protein members (Additional file 10) are common to the three conifer species, and were annotated as containing “pentatricopeptide -PPR- repeats”, either implicated in *replication, recombination and repair* or with a *function unknown*, “terminal inverse repeats -TIR-”, “leucine rich repeats” or “NB-ARC domains”, which are molecular switches implicated in *signal transduction mechanisms*. On the other hand, the groups exclusive of *P. pinaster* that include at least 10 proteins were annotated as “zinc finger proteins” (19 members), “sister chromatid cohesion protein PDS5” (13 members), “GDP-L-galactose phosphorylases” and “zinc ion binding proteins” (11 members each), and several clusters annotated as “retrotransposon proteins”. However, most of the groups exclusive of *P. pinaster* contained only one protein.

**Fig. 2:**
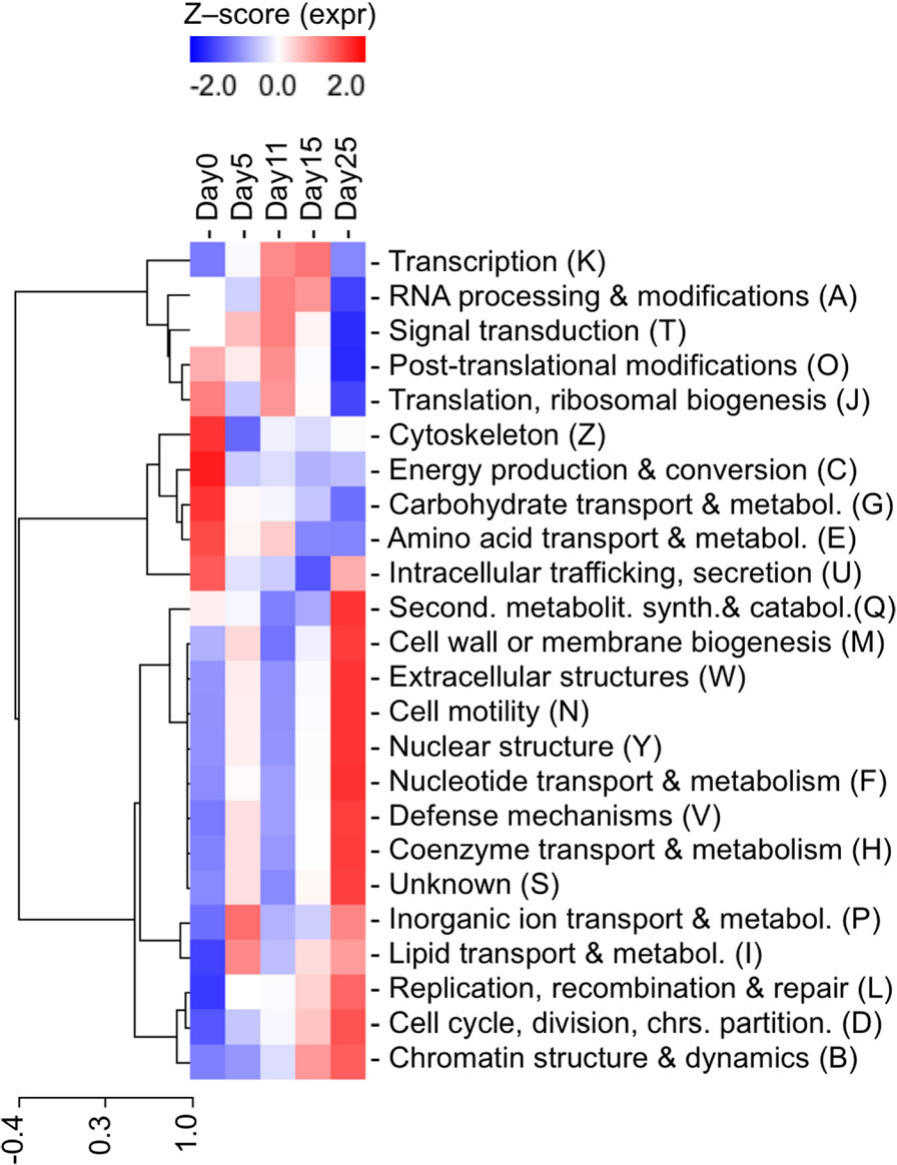
Heatmap of the eggNOG functional categories expressed throughout *P. pinaster* embryo developmental stages. The heatmap was built after the counts of each transcript belonging to a protein group in such category were added up, and later transformed in Z-scores. The capital letters in between brackets originate in the eggNOGs database and are specific to the functional categories. The hierarchical clustering on the left side of the image represents the correlation of the expression levels associated with distinct functional categories

Since each protein group was classified into a functional eggNOG annotation [35], it was possible to condense the functional information to 24 categories. Most of the predicted proteins and associated eggNOGs had *function unknown* (5684 from the total 14,134 eggNOGS found in *P. pinaster*) (Additional file 10). For the remaining, a heatmap of functional categories expressed throughout *P. pinaster* embryo development (Fig. 2) shows three major clusters of functional categories predominantly expressed at the early (Day0), middle (Day5 and Day11) or late (Day15 and Day25) embryogenesis stages. At the early embryo stages, functions associated to the *cytoskeleton*, *energy production and conversion*, *carbohydrate transport and metabolism*, *amino acid transport and metabolism* and *intracellular trafficking, secretion and vesicular transport* are prevalent, with the first three being just up-regulated at these stages. In contrast, *replication, recombination and repair*, and *cell cycle control, cell division, chromosome partitioning*, and *chromatin structure and dynamics* follow the opposite trend and are up-regulated in late embryo stages.

Regarding the overall metabolic activity of the developing embryo, *carbohydrate transport and metabolism* is upregulated at early embryo stages decreasing towards the mature embryo, *amino acid transport and metabolism* is predominant in early embryo to early cotyledonary embryo stages, while *secondary metabolites biosynthesis, transport, and catabolism* peak at the mature embryo stage. *Nucleotide transport and metabolism* and *coenzyme transport and metabolism* show highest expression at mature embryo stage, whereas *lipid transport and metabolism* is relevant both in the pre-cotyledonary and mature embryo stages.

### Differentially expressed transcripts along embryo development

From the 67,429 assembled transcripts, 64,766 are clearly expressed in at least one developmental stage and 39,838 are expressed in all five stages (Additional file 11). The number of transcripts expressed in each stage is equivalent, over 10,000 expressed transcripts per stage. A total of 4953 transcripts (7.3%) are expressed in only one stage of embryo development: 848, 338, 282, 1703, 1782 are exclusively expressed in Day0, Day5, Day11, Day15, and Day25, respectively.

**Fig. 3:**
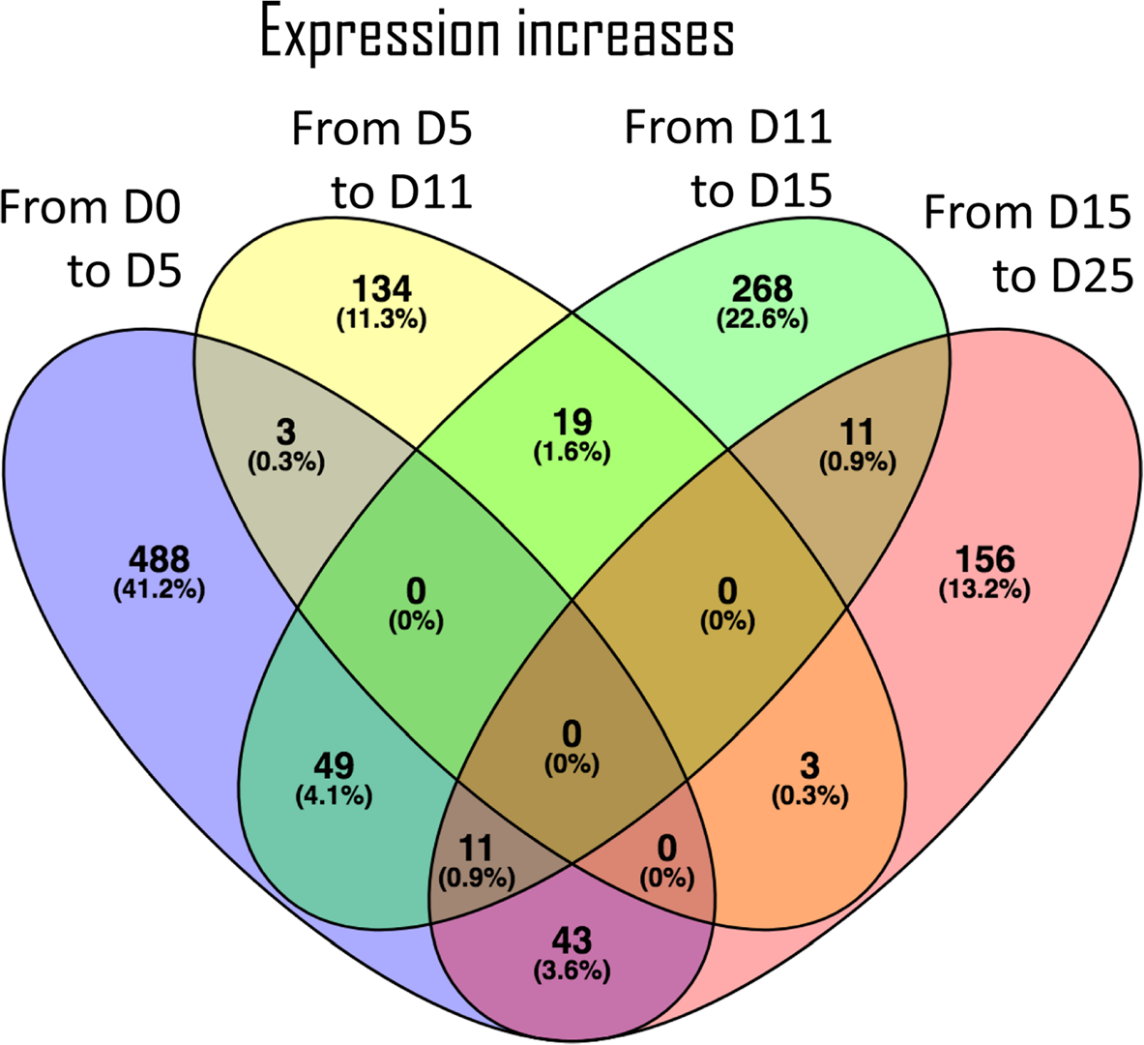
Venn diagram of the 1185 differentially expressed transcripts found up-regulated between two consecutive embryo developmental stages. The number of transcripts and respective percentage (relative to the total aforementioned 1185 transcripts) are represented for each transition. The numbers in the intersections represent transcripts found up-regulated in more than one developmental transition

**Fig. 4:**
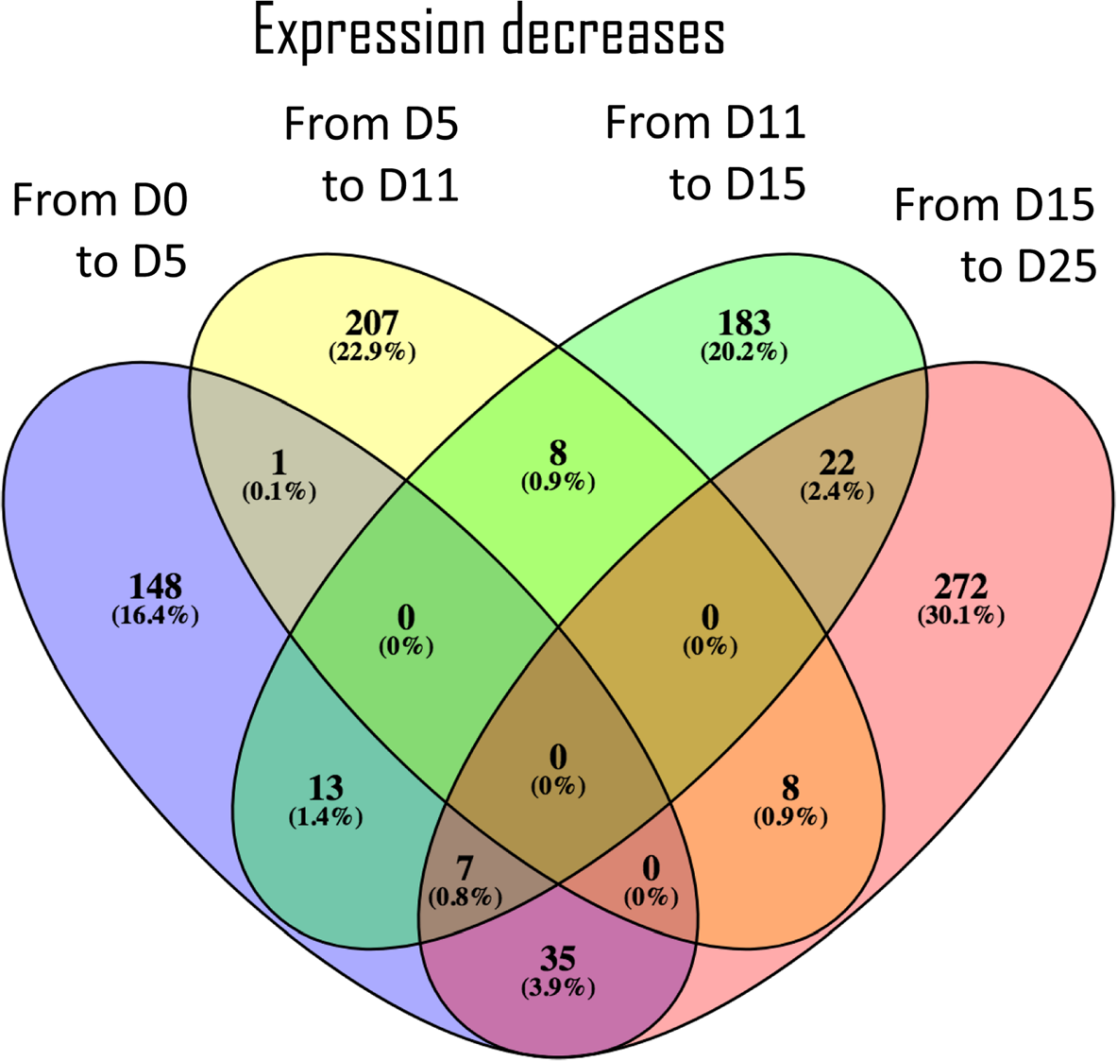
Venn diagram of the 904 differentially expressed transcripts found down-regulated between two consecutive embryo developmental stages. The number of transcripts and respective percentage (relative to the total aforementioned 904 transcripts) are represented for each transition. The numbers in the intersections represent transcripts found down-regulated in more than one developmental transition

A differential expression analysis (FDR < 0.05) between each pair of consecutive stages identified 1738 transcripts (2.6%) differentially expressed in at least one transition (Additional file 12). A total of 798, 383, 591 and 568 transcripts were differentially expressed in the first (from Day0 to Day5), second (from Day5 to Day11), third (from Day11 to Day15), and fourth (from Day15 to Day25) transition, respectively. Up-regulation of differentially expressed transcripts is predominant in the first transition, accounting for 594 of the 1185 up-regulated differentially expressed transcripts (Fig. 3) specific for first transition, while down-regulation of differentially expressed transcripts is more abundant in the last transition (Fig. 4), representing approximately 38% of the total down-regulated transcripts. Each developmental transition shares few up- or down-regulated transcripts with the consecutive transition, supporting that the developmental stages selected for this study are clearly differentiated in terms of ongoing transcriptional activity.

A gene enrichment analysis of the differentially expressed transcripts retrieved over-represented GO terms associated with the first and last stage transitions (see Additional files 13, 14 and 15). Both transitions share an enrichment of down-regulated transcripts associated with *regulation of cell cycle* and *cell division*. Moreover, the first transition seems to be characterized by down-regulation of transcripts involved in *monosaccharide transport* and *plant-type cell wall cellulose metabolism*. The last transition is characterized by down-regulated transcripts related with epigenetics and annotated with the *DNA-dependent DNA replication* GO term. As for the up-regulated transcripts, those found in the first transition are associated with *terpenoid catabolism* and *tertiary alcohol metabolism*, while the only enriched GO term found in last transition is the molecular function *nutrient reservoir activity* (data not shown). Overall, the terms carbohydrate transport and metabolism (Table 3) and epigenetics related terms (Table 4) are highly represented in the list of differentially expressed transcripts.

**Table 3:** Differentially expressed transcripts related to carbohydrate transport and metabolism

Cluster^a^	Transcript	*At* Locus	*Pta* Locus	*Pla* Locus	Annotation^b^
k1	Pp10265	#N/A	#N/A	#N/A	phosphoenolpyruvate carboxykinase
k1	Pp1126	#N/A	#N/A	#N/A	mannan endo- -beta-mannosidase
k1	Pp19154	#N/A	#N/A	#N/A	cytosolic triosephosphate isomerase
k1	Pp28580	AT4G37870.1	2A_all_VO_L_6958_T_66/101|m.15610	V1_2kb/polished_high/000086|m.144	phosphoenolpyruvate carboxykinase
k1	Pp42692	#N/A	#N/A	#N/A	udp-d-glucuronate 4-epimerase 2
k1	Pp45907	#N/A	#N/A	#N/A	triose phosphate phosphate non-green precursor
k1	Pp45908	#N/A	#N/A	#N/A	phosphate phosphoenolpyruvate translocator precursor
k2	Pp11437	#N/A	#N/A	#N/A	xyloglucan endotransglucosylase hydrolase
k2	Pp11438	#N/A	#N/A	P/miseq/c27580_g1_i1|m.27950	PREDICTED: probable xyloglucan endotransglucosylase/hydrolase protein 26
k2	Pp12819	AT1G76160.1	2A_all_VO_L_1_T_165541/181832|m.14942	E1_2kb_2/polished_high/000754|m.928	L-ascorbate oxidase homolog SKU5 SIMILAR 5 (SKS5)
k2	Pp1567	#N/A	#N/A	#N/A	multicopper oxidase
k2	Pp18330	#N/A	6A_all_VO_L_6290_T_21/157|m.53348	#N/A	PREDICTED: sugar transport protein 13
k2	Pp18567	#N/A	#N/A	DCR/hiseq/c107286_g1_i1|m.126449	PREDICTED: probable pectinesterase 68
k2	Pp1892	#N/A	#N/A	#N/A	aldose 1-epimerase
k2	Pp20813	#N/A	#N/A	#N/A	hexose transporter
k2	Pp2456	#N/A	#N/A	#N/A	cellulose synthase
k2	Pp27962	#N/A	5A_I15_VO_L_1793_T_10/15|m.46809	#N/A	PREDICTED: CMP-sialic acid transporter 2
k2	Pp28117	#N/A	#N/A	#N/A	alcohol dehydrogenase
k2	Pp28964	#N/A	2A_all_VO_L_8976_T_26/41|m.15937	#N/A	Xyloglucan endotransglucosylase/hydrolase protein A precursor, putative
k2	Pp30302	#N/A	#N/A	#N/A	glyoxalase i
k2	Pp32670	AT5G26340.1	#N/A	JASS/hiseq/c63525_g1_i1|m.64429	SUGAR TRANSPORT PROTEIN 13 (STP13), ATSTP13, MSS1
k2	Pp32960	#N/A	#N/A	#N/A	probable inositol transporter 2-like
k2	Pp34009	#N/A	3A_I18_VO_L_2_T_2368/136250|m.32448	Basket/c19128_g1_i1|m.26924	PREDICTED: L-ascorbate oxidase homolog
k2	Pp36526	#N/A	#N/A	#N/A	L-ascorbate oxidase-like protein
k2	Pp37397	#N/A	#N/A	#N/A	nadp-dependent malic enzyme
k2	Pp37548	AT4G39770.1	#N/A	M_S1/c25586_g1_i4|m.129341	trehalose-6-phosphate phosphatase
k2	Pp38427	#N/A	#N/A	#N/A	pyruvate kinase
k2	Pp38450	AT2G01850.1	2A_I15_VO_L_1_T_124932/133144|m.21738	V1_1kb_1/015976|m.720027	ENDOXYLOGLUCAN TRANSFERASE A3 (EXGT-A3), XYLOGLUCAN ENDOTRANSGLUCOSYLASE/HYDROLASE 27 (XTH27), ATXTH27
k2	Pp38781	AT4G02290.1	2A_I15_VO_L_39_T_24/48|m.21781	S_2kb/polished_high/000346|m.477	glycosyl hydrolase 9B13, endoglucanase 17
k2	Pp38924	#N/A	#N/A	#N/A	xylose isomerase
k2	Pp39507	AT5G13870.1	6A_I20_VO_L_1_T_64241/133533|m.55411	E1_2kb_2/polished_high/002288|m.2304	ENDOXYLOGLUCAN TRANSFERASE A4 (EXGT-A4), XYLOGLUCAN ENDOTRANSGLUCOSYLASE/HYDROLASE 5 (XTH5)
k2	Pp42916	#N/A	5A_I12_VO_L_2_T_51143/53062|m.43159	#N/A	phosphoenolpyruvate carboxykinase
k2	Pp43330	#N/A	#N/A	#N/A	pectin methylesterase (pectinesterase)
k2	Pp43761	#N/A	#N/A	#N/A	glycosyl hydrolase-like protein
k2	Pp46170	AT3G13790.1	6A_all_VO_L_6326_T_78/89|m.53354	#N/A	*Arabidopsis thaliana* CELL WALL INVERTASE 1 (ATCWI1), ATBFRUCT1, ATCWINV1, CWI1
k2	Pp47826	AT5G03630.1	5A_all_VO_L_2839_T_31/70|m.40851	Wound/hiseq/c70186_g2_i1|m.47659	Pyridine nucleotide-disulphide oxidoreductase family protein (MDAR2), monodehydroascorbate reductase
k2	Pp6019	AT1G77210.1	#N/A	#N/A	SUGAR TRANSPORT PROTEIN 14 (STP14), ATSTP14
k2	Pp6337	#N/A	#N/A	#N/A	multicopper oxidase
k2	Pp8252	#N/A	#N/A	#N/A	hexose transporter
k2	Pp8434	AT1G68560.1	#N/A	S_2kb/polished_high/000741|m.810	ALPHA-XYLOSIDASE 1 (XYL1), ALTERED XYLOGLUCAN 3 (AXY3), THERMOINHIBITION RESISTANT GERMINATION 1 (TRG1), ATXYL1, GH31
k2	Pp8435	#N/A	#N/A	#N/A	alpha-xylosidase precursor
k2	Pp8535	#N/A	#N/A	#N/A	endoglucanase
k2	Pp9898	AT3G59480.1	#N/A	S_2kb_2/020091|m.629185	pfkB-like carbohydrate kinase family protein
k3	Pp15644	#N/A	#N/A	#N/A	beta glucosidase 43
k3	Pp21641	#N/A	#N/A	#N/A	mannan endo- -beta-mannosidase 7
k3	Pp23792	#N/A	6A_all_VO_L_13474_T_9/19|m.54026	V1_1kb_3/polished_high/001086|m.1085	senescence-associated protein 29
k4	Pp16423	#N/A	#N/A	#N/A	protein
k4	Pp24290	#N/A	#N/A	#N/A	beta-amylase 7-like
k4	Pp27124	#N/A	#N/A	#N/A	o-glycosyl hydrolases family 17 protein
k4	Pp34845	#N/A	#N/A	#N/A	phosphatidylinositol 4-kinase
k4	Pp9901	#N/A	#N/A	#N/A	alkaline alpha galactosidase i
k5	Pp14379	#N/A	#N/A	#N/A	O-Glycosyl hydrolases family 17 protein
k5	Pp1469	#N/A	#N/A	#N/A	unknown; unknown [*Picea sitchensis*]
k5	Pp32140	#N/A	#N/A	#N/A	brassinosteroid-regulated protein bru1
k5	Pp32141	#N/A	#N/A	SDN/miseq/c29601_g2_i2|m.39898	xyloglucan endotransglucosylase/hydrolase protein 24
k5	Pp32144	#N/A	#N/A	P/miseq/c33524_g1_i3|m.50829	PREDICTED: probable xyloglucan endotransglucosylase/hydrolase protein 23-like
k5	Pp39583	#N/A	#N/A	#N/A	thermostable beta-glucosidase
k5	Pp41586	#N/A	#N/A	#N/A	probable glycerophosphoryl diester phosphodiesterase 3-like
k5	Pp4948	#N/A	#N/A	#N/A	catalase
k5	Pp7568	#N/A	#N/A	#N/A	alcohol dehydrogenase
k5	Pp9008	AT1G22170.1	#N/A	E1/hiseq/c25131_g1_i3|m.5975	Phosphoglycerate mutase-like protein
k6	Pp23909	#N/A	#N/A	#N/A	mannan endo- -beta-mannosidase 7
k6	Pp876	#N/A	#N/A	#N/A	neurofilament protein h form h2
k6	Pp9495	#N/A	#N/A	#N/A	phospholipase c
k7	Pp26503	#N/A	#N/A	#N/A	galactinol synthase
k7	Pp2858	#N/A	#N/A	#N/A	succinate dehydrogenase
k7	Pp34900	#N/A	#N/A	#N/A	malate synthase
k7	Pp34906	AT5G03860.1	2A_I2_OT_comp27109_c0_seq3|m.23989	Neg_S1/c31207_g1_i2|m.108321	malate synthase
k7	Pp40679	#N/A	6A_I23_VO_L_1_T_44295/165398|m.58874	BRN/hiseq/c65594_g2_i2|m.37248	galactinol synthase
k7	Pp40719	AT3G24090.1	5A_all_VO_L_2_T_192437/409051|m.39335	E1/hiseq/c40068_g1_i1|m.27499	glucosamine-fructose-6-phosphate aminotransferase
k8	Pp14503	#N/A	#N/A	#N/A	beta-amylase 7-like
k8	Pp44718	#N/A	#N/A	#N/A	beta-amylase 7-like
k9	Pp32321	#N/A	#N/A	P/miseq/c26472_g1_i4|m.24888	endoxyloglucan transferase A4
k9	Pp39715	#N/A	3A_I18_VO_L_2_T_63095/136250|m.32723	#N/A	aldose 1-epimerase family protein
k9	Pp40720	#N/A	#N/A	#N/A	glucosamine--fructose-6-phosphate aminotransferase
k10	Pp240	#N/A	#N/A	#N/A	beta-glucosidase 44-like
k10	Pp30121	AT4G38970.1	3A_I16_VO_L_2_T_48696/60890|m.30958	V_1kb/017920|m.889708	fructose-bisphosphate aldolase 2
k11	Pp14541	#N/A	#N/A	#N/A	aldehyde dehydrogenase family 2 member mitochondrial-like
k11	Pp15288	#N/A	#N/A	#N/A	pyruvate decarboxylase isozyme
k11	Pp18297	#N/A	5A_I14_VO_L_947_T_33/56|m.45171	DCS1kb_1/003053|m.3985	PREDICTED: probable xyloglucan endotransglucosylase/hydrolase protein 8-like
k11	Pp23089	#N/A	#N/A	#N/A	polygalacturonase
k11	Pp35326	#N/A	#N/A	#N/A	protein
k11	Pp9001	#N/A	#N/A	#N/A	probable xyloglucan endotransglucosylase hydrolase protein 23
k11	Pp995	#N/A	#N/A	#N/A	myo-inositol-1-phosphate synthase
k11	Pp9981	AT1G32860.1	#N/A	#N/A	Glycosyl hydrolase superfamily protein
k12	Pp46622	AT4G25000.1	#N/A	SDN/miseq/c15856_g1_i1|m.11496	alpha-amylase-like

^a^Number of the cluster of expression that the transcript is associated with

^b^Order of preference for annotating each *P. pinaster* transcript after its homologs is: *A. thaliana*, *P. taeda*, *P. lambertiana*, and Blast2Go annotation against NCBI

**Table 4:** Differentially expressed transcripts involved in epigenetic regulation (with BRH found)

Cluster^a^	Transcript	*At* Locus	*Pta* Locus	*Pla* Locus	Annotation^b^
DNA modification					
3	Pp11214	AT1G57820.1	#N/A	V_2kb_2/polished_high/000175|m.181	VARIANT IN METHYLATION 1 (VIM1), ORTHRUS 2 (ORTH2)
5	Pp3794	#N/A	#N/A	P/miseq/c15561_g1_i1|m.8297	PREDICTED: DNA (cytosine-5)-methyltransferase DRM2-like
Histone modification					
1	Pp30887	#N/A	#N/A	SDN/miseq/c40928_g1_i1|m.61118	ubiquitin-conjugating enzyme 28, E2
3	Pp44003	AT5G24330.1	6A_all_VO_L_4227_T_27/112|m.53170	DCS/hiseq/c48519_g1_i1|m.18331	ARABIDOPSIS TRITHORAX-RELATED PROTEIN 6 (ATXR6), SET DOMAIN PROTEIN 34 (SDG34)
10	Pp33894	#N/A	5A_I16_NT_comp45588_c0_seq2|m.47551	S/hiseq/c42737_g1_i1|m.36406	PREDICTED: histone-lysine N-methyltransferase SUVR5
Chromatin formation or chromatin remodelling					
3	Pp26994	AT5G22750.1	#N/A	S/hiseq/c37932_g2_i1|m.21712	RAD5, RAD5A
3	Pp34781	AT1G65470.1	6A_I23_VO_L_4689_T_39/51|m.59955	RF-S_3/polished_high/005053|m.5101	FASCIATA 1 (FAS1), FUGU 2, FUGU2, NFB2, NUCLEOSOME/CHROMATIN ASSEMBLY FACTOR GROUP B
7	Pp30270	AT5G37055.1	#N/A	BRN/hiseq/c66142_g2_i1|m.38827	SERRATED LEAVES AND EARLY FLOWERING (SEF), ATSWC6
8	Pp14163	AT1G05490.1	#N/A	S/hiseq/c33164_g1_i2|m.13589	CHROMATIN REMODELING 31 (CHR31)
RNA silencing					
1	Pp12441	#N/A	5A_I13_OT_comp19461_c0_seq4|m.44088	#N/A	PREDICTED: protein argonaute 2-like
Histones					
3	Pp36206	#N/A	#N/A	E1_2kb_2/polished_high/001369|m.1490	histone H3
3	Pp38724	#N/A	#N/A	DCS1kb_3/polished_high/000803|m.897	histone H2A 12
6	Pp46360	#N/A	2A_all_VO_L_1_T_65055/181832|m.14308	#N/A	PREDICTED: histone H1-like
10	Pp14332	AT2G30620.1	#N/A	M_S1/c4520_g1_i1|m.114500	HISTONE 1.2 (H1.2)
10	Pp46359	#N/A	#N/A	SDN/miseq/c31005_g5_i1|m.46415	histone H1.2
Others					
3	Pp41359	#N/A	5A_all_VO_L_69333_T_11/13|m.42398	V_3_3-6 kb/016515|m.360307	Telomere-associated protein RIF1

^a^Number of the cluster of expression that the transcript is associated with

^b^Order of preference for annotating each *P. pinaster* transcript after its homologs is: *A. thaliana*, *P. taeda*, and *P. lambertiana*

### Clustering of the differentially expressed transcripts

**Fig. 5:**
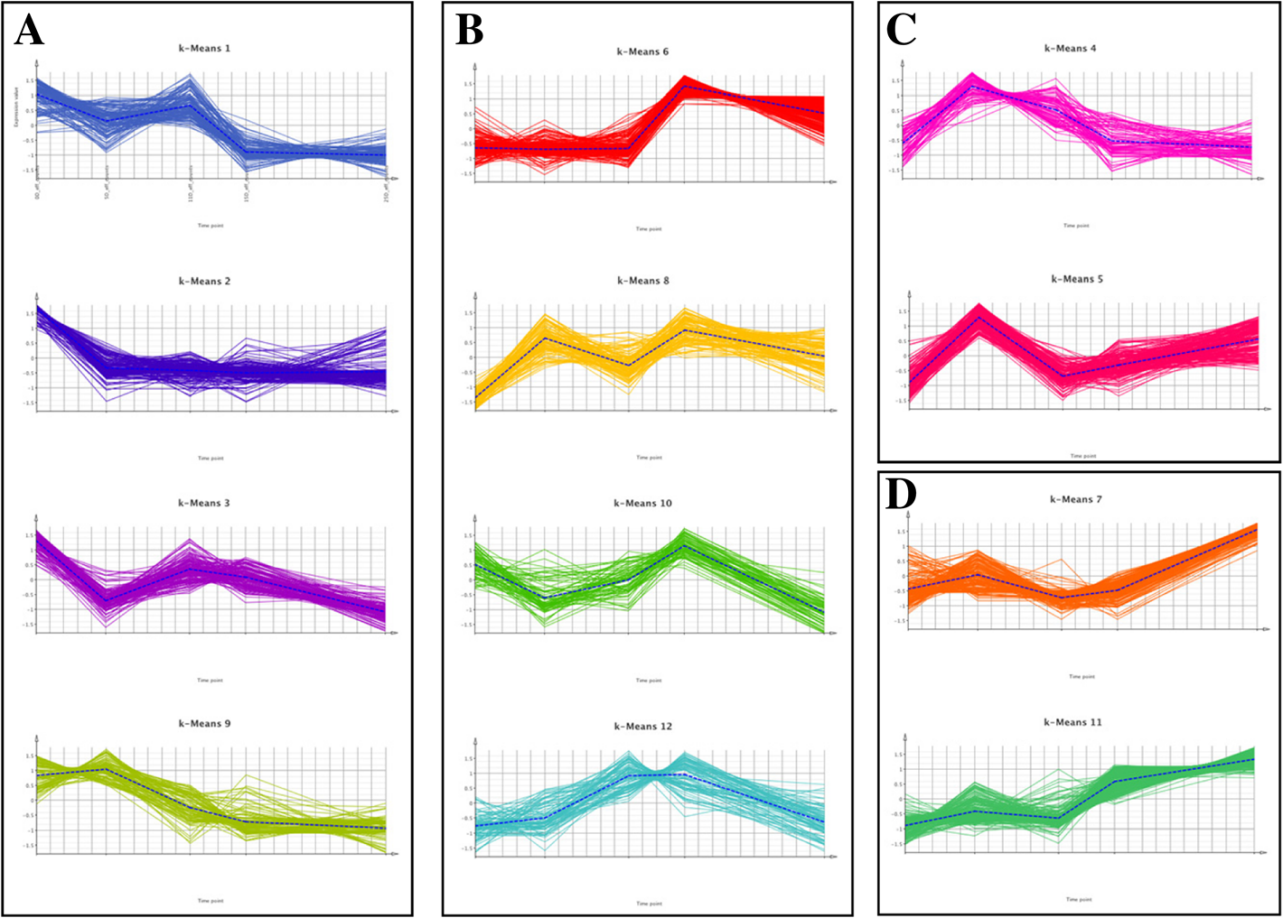
K-means clustering of differentially expressed transcripts along *P. pinaster* embryo development. Transcripts were clustered together according to their expression profiles and a representative mean expression profile (dashed line) was represented for every cluster. The 12 k-means clusters generated were further divided in four groups (A to D) depending on the embryo developmental stage in which the expression peaks. The inset displays the processes and functions found over-represented among the transcripts from the different clusters (and groups). **a** Clusters K1, K2, K3, K9, showing decreasing expression along development. Cellular carbohydrate metabolism, monosaccharide transport, carbohydrate metabolism, sterol metabolism, cell wall organization or biogenesis, external encapsulating structure organization,cell wall macromolecule metabolism, DNA-dependent DNA replication, biological regulation, organic cyclic compound metabolism, cellular aromatic compound metabolism, cellular component organization or biogenesis, cell cycle,developmental process, shoot system development, multicellular organismal process, mitotic cell cycle process, methylation, cell proliferation, microtubule-based process. **b** Clusters K6, K8, K10, K12, with expression peaking at Day11 and/or Day15. Steroid dehydrogenase activity, acting on the CH-CH group of donors, 3-oxo-5-alpha-steroid 4-dehydrogenase activity, abscisic acid catabolism, release of seed from dormancy. **c** Clusters K4, K5, with expression peaking at Day5. No GO-terms. **d** Clusters K7, K11, with increasing expression along development. Nutrient reservoir activity, carbon-oxygen lyase activity, acting on phosphates, carbon-oxygen lyase activity, alpha-bisabolene synthase activity, terpene synthase activity

The 1738 differentially expressed transcripts throughout embryo development could be grouped into 12 distinct clusters (K) with similar patterns of expression by k-means clustering analysis (Fig. 5). Overall, the clusters could be further divided in four groups (A to D) according to the embryo stage in which the expression peaks: (A) early embryo stages (clusters 1, 2, 3, 9), (B) early-cotyledonary and/or cotyledonary stages (clusters 6, 8, 10, 12), (C) pre-cotyledonary embryo stages (clusters 4, 5), and (D) mature embryo stage (clusters 7, 11). Following an enrichment analysis performed in each cluster, several processes and functions were found over-represented in different phases of embryo development (Fig. 5). The transcripts in group A are enriched in several GO terms including *carbohydrate metabolism*, *monosaccharide transport*, *sterol metabolism*, *cell wall organization or biogenesis*, *DNA-dependent DNA replication*, *cell cycle*, *regulation of gene expression, epigenetic*, among others (Fig. 5). In cluster 2, processes related to *carbohydrate metabolism* and *monosaccharide transport* and to the cell wall are highly represented (Additional file 16). In cluster 3, which differs from cluster 2 mainly because the expression stays relatively high at mid-embryo stages before decreasing towards the mature embryo stage, *DNA-dependent DNA replication* is particularly relevant in terms of the number of contributing transcripts (89 transcripts) and includes *chromatin organization*, *DNA modification*, *DNA methylation*, *histone lysine methylation*, *DNA packaging*, *histone methylation*, *regulation of gene expression, epigenetic* (Additional file 17). The transcripts in group B are enriched in GO terms such as *steroid dehydrogenase activity*, *sesquiterpenoid catabolism, release of seed from dormancy* and *tertiary alcohol metabolism*. In group D, the most represented terms are *nutrient reservoir activity*, *carbon-oxygen lyase activity*, *alpha-bisabolene synthase activity* and *terpene synthase activity*.

### Validation by qPCR

**Fig. 6:**
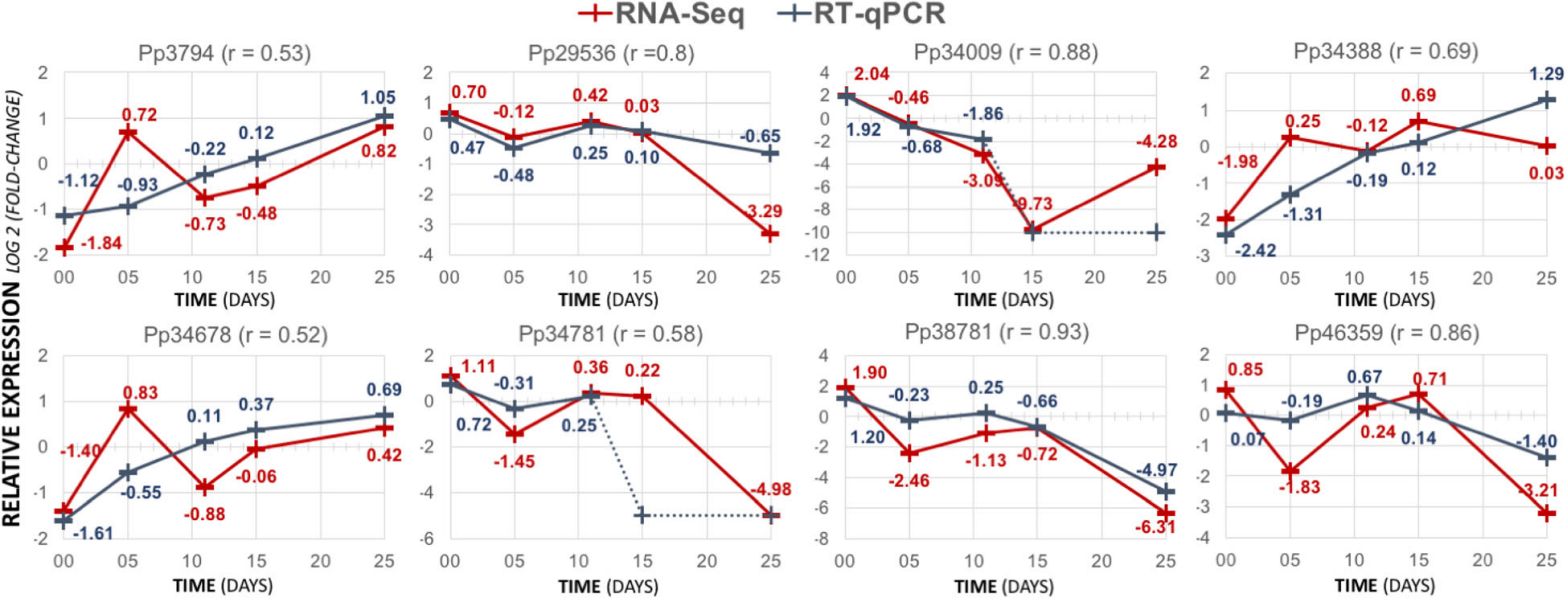
Validation of *P. pinaster* transcripts profile obtained by RNA-seq (red line) with RT-qPCR (blue line). Pearson correlation values (r) between the two technologies are shown. Fold-change values are also shown for each developmental stage. Dotted lines connect relative expression values calculated for Cq values detected in the last 5 cycles of the qPCR amplification program

A subset of eight transcripts was selected based on their expression profile and putative involvement in carbohydrate metabolism (Table 3) or epigenetic regulation (Table 4), to independently validate the RNA-seq results using RT-qPCR (information about the primers can be found in Additional file 18). Five of these genes are differentially expressed transcripts throughout embryo development (FDR < 0.05) and are included in different transcription profiles (clusters 2, 3, 5, 10). The RT-qPCR and RNA-seq expression results are generally in good agreement as demonstrated by the values of Pearson correlation, which ranged between 0.52 (Pp34678) and 0.93 (Pp38781), confirming the peaks of expression associated with specific developmental stages (Fig. 6). There is also a good agreement between RT-qPCR and RNA-seq for transcripts which had not been predicted as differentially expressed (Pp29536, Pp34388 and Pp34678).

## Discussion

In this work, RNA-seq is used to provide a comprehensive overview of the transcriptome of the maritime pine developing embryo. Previously, the first transcriptomic analysis of the maritime pine embryo using the same developmental stages as those analysed here, has relied on the use of a loblolly pine (*P. taeda*) microarray to identify transcripts present during embryogenesis which are conserved between the two species [36]. Although the microarray had been successfully used for cross-species hybridization [37, 38], it contained approximately 25,000 unique cDNAs from *P. taeda* representative of cDNA libraries exclusively prepared from root and needle tissues, limiting the scope of the analysis considering our main focus on embryo development. In the present study, such limitation is overcome and the transcriptomic landscape during embryogenesis has been significantly expanded. Over 300 M read-pairs obtained from embryos at five stages of development were assembled using a comprehensive strategy to produce a reference transcriptome for maritime pine zygotic embryogenesis with 67,429 transcripts. This number is three times the number of expressed transcripts that had been previously identified with the *P. taeda* microarray [36], corresponding to a similar increase in the number of annotated proteins. Nevertheless, *Picea sitchensis*, *Vitis vinifera* and *Ricinus communis* remain the top three most represented species in the distribution of all Top-BLAST hits.

The annotation of *P. pinaster*, *P. taeda* and *P. lambertiana* predicted proteins with the eggNOG number of its best homolog revealed that most annotated proteins are shared by the three species (40.3% in *P. pinaster*, 51.1% in *P. lambertiana*, and 86.2% in *P. taeda*). *P. pinaster* and *P. lambertiana* predicted proteomes have approximately 10 times more proteins in common than those shared with *P. taeda*, while the latter shares roughly the same percentage of proteins with either species. Moreover, the percentage of exclusive predicted proteins was higher in *P. pinaster* (30.8%), against 3.0% in *P. taeda*. The analysis of orthologous groups highlighted some eggNOGs, shared by the three species, with a high number of protein members, which include “PPR repeat” and “Retrotransposon protein” involved in *replication, recombination and repair*, or with *function unknown*, and several eggNOGs described as “Leucine Rich Repeat (LRR)”, and “nucleotide binding domain with an ARC motif (NB-ARC domain)” involved in *signaling transduction mechanisms*, and “toll/interleukin-1 receptor (TIR)” with *function unknown*. These results are in close agreement with the reported annotation of the shoot transcriptome of *Pinus patula* in which the largest family that was identified, including 1794 members, contained LRR, TIR, NB-ARC, Golgi transport complex 5 (COG5) and poxvirus A32 protein motifs [31].

### Carbohydrate metabolism and transport in early embryogenesis

This work gathered several evidences, both from the analysis of functional categories represented throughout embryo development and from the analysis of specific transcripts, pointing to a prominent role of carbohydrate transport and metabolism early in *P. pinaster* embryogenesis. The analysis of functional categories assigned to eggNOGs showed up-regulation of *carbohydrate transport and metabolism* in early embryo stages and a reduction in expression towards later stages of embryogenesis. Also, the enrichment analysis of all the differentially expressed transcripts revealed a group of highly expressed transcripts peaking at Day0 (cluster 2 profile) enriched in *carbohydrate metabolism*, including *cellular carbohydrate metabolism*. In the same profile, *cellular carbohydrate metabolism* appears associated to the metabolism of the primary cell wall components cellulose, pectin, and glucan. Additionally, the 204 differentially expressed transcripts down-regulated from Day0 to Day5 were enriched in *monosaccharide transport* and *plant-type cell wall cellulose metabolism* (see Additional file 13). A recent study in *P. sylvestris* seed development has also pointed out to an over-representation of *carbohydrate metabolic process* and *cell wall modification* terms among the differentially expressed transcripts over-represented at early developmental stages [21].

Many glycoside hydrolases encoding transcripts were found more expressed in the first embryo stage (cluster 2), including the Pp38781 homolog of AT4g02290 (glycosyl hydrolase 9 family) whose expression profile was successfully validated by RT-qPCR. In particular, the presence of several pine homologs of α-xylosidase and xyloglucan endotransglycosylase (previously named xyloglucan-endo-β-glucanase) within this expression profile points out the importance of xyloglucan mobilization and/or degradation in pine early embryogenesis [39]. Xyloglucan is the main hemicellulose constituent of the primary cell walls of spermatophytes except for grasses (reviewed by [40]), and the glycosidases capable of trimming the xyloglucan side chains are expected to act in the turnover or recycling of xyloglucan during cell wall expansion (reviewed in [41]). The Arabidopsis *ALPHA-XYLOSIDASE 1/ ALTERED XYLOGLUCAN 3/ THERMOINHIBITION RESISTANT GERMINATION 1 (XYL1/ AXY3/ TRG1)*, whose putative maritime pine homolog (Pp8434) was also up-regulated in early embryogenesis, codes for an enzyme involved in xyloglucan degradation into free monosaccharides [42] and the only α-xylosidase active against xyloglucan [43]. Germinating seeds of Arabidopsis *xyl1* loss-of-function mutants display cell wall loosening and reduced seed dormancy due to alterations in primary cell wall integrity [44]. Also a putative homolog (Pp38450) of the Arabidopsis *ENDOXYLOGLUCAN TRANSFERASE A3/ XYLOGLUCAN ENDOTRANSGLUCOSYLASE/HYDROLASE 27* (*EXGT-A3/ XTH27*), involved in the differentiation of tracheary elements through the degradation of the xyloglucan deposited in the cell walls [45], was identified with the same expression profile (cluster 2), together with a putative homolog of *XTH5,* a gibberellin (GA)-inducible gene expressed in the embryonic axis and in the radicle of seeds, involved in germination [46]. Overall, it seems that the glycoside hydrolases involved in cell-wall modification during germination, are also relevant for the control of early stages of pine embryogenesis. In fact, enzymes responsible for xyloglucan degradation have already been found associated to somatic embryogenesis induction in divergent species (reviewed by [47, 48]), including conifers such as *P. radiata* where the up-regulation of α-D-galactosidase (SEPR1) was detected [49]. In *Picea abies* somatic embryogenesis a *Xyloglucan:xyloglucosyl transferase* and a *Glycosyl transferase family 1 protein* were found differentially expressed and down-regulated in the transitions from proliferation to differentiation of early embryos and from early embryos to development of late embryos [38].

Other members of the glycosyl hydrolase family were found differentially expressed in our results, including a putative homolog (Pp46170) of *CELL WALL INVERTASE 1* (*CWINV1/CWI1*), described as playing a role in plant sink tissues where it performs the hydrolysis of sucrose, unloaded from the phloem via apoplast, into fructose and glucose (reviewed in [50]). Studies in cotton and Arabidopsis seeds revealed that *CWINV* is expressed all over the globular embryo but seems to be restricted to the central zone of the torpedo embryo [51]. In a comparison of the response to somatic embryogenesis induction in shoot primordia derived from adult trees of *Picea glauca*, up-regulation of *CWINV1* was detected only in non-responsive genotype, and a possible link with biotic stress response was discussed [52]. There are also many evidences that associate CWINV-mediated sucrose hydrolysis with the sugar signaling that promotes cell division in early embryogenesis (reviewed by [50, 53]).

Many transcripts coding for putative carbohydrate transporters also peaked at the first embryo stage (Day0), most of them hexose (or monosaccharide) transporters involved in transport and intake into the cytoplasm, possibly after CWIN has cleaved the sucrose present in the apoplast [51]. Although still very little is known about the role played by these hexose transporters specifically in early plant embryogenesis (reviewed in [54]), previous work on other sink tissues that, like the developing embryo, are also symplastically isolated, had shown their dependence on transport proteins to uptake the sucrose-derivatives through the plasma membrane (reviewed by [55]). A putative pine homolog (Pp6019) of the Arabidopsis SUGAR TRANSPORT PROTEIN 14 (*AtSTP14*) belonging to the AtSTPs family, but specific for galactose transport and expressed both in source (green leaves) and in sink tissues (seed endosperm and cotyledons) [56], was also detected. There are several evidences pointing to a role of AtSTP14 in cell wall recycling, namely in the transport of the cell wall-derived galactose released upon cell wall degradation performed by β-galactosidases (glycosyl hydrolases) and other enzymes [56]. Yet another putative pine homolog (Pp32670) of the Arabidopsis *AtSTP13* was found in cluster 2, likely involved in the transport of monosaccharides derived from the cell wall (reviewed in [54]).

The GO enrichment results from clusters 2 and 3, which gather several glycosyl hydrolases, carbohydrate transporters and kinases, as well as cell cycle related transcripts, seem to support an active communication between regulators of carbohydrate metabolism and cell cycle in *P. pinaster* early embryogenesis. So far, the impact on plant cell cycle control and cell division derived from the interaction between the nutritional state and genetic control has been elucidated only in post-embryonic development studies [57]. Observations in Arabidopsis show that carbohydrates availability plays a role on cell decision over G2/M transition by sugar signaling interaction with specific key cell cycle regulators, such as CYCB1;1 and CDKB1;1, which directly impacts proliferation of meristematic tissues [58]. Among the five *cyclin dependent kinase b* transcripts found among clusters 2 and 3 there is Pp42651, a putative pine homolog of the Arabidopsis CYCLIN-DEPENDENT KINASE B2;2 (CDKB2;2) which has been shown to be a regulator of cell cycle progression and SAM organization, and involved in hormone signaling [59].

### Epigenetics associated transcripts in early to middle embryogenesis

Many transcripts associated with different components of epigenetic regulation are found differentially expressed during maritime pine embryogenesis. Most of them are included in cluster 3 profile, characterized by a peak of expression at early embryogenesis.

Five putative histone subunits homologs are differentially expressed across pine embryo development and follow different expression profiles. Pp36206 and Pp38724, putatively encoding core H3 and H2A histone subunits, respectively, show a higher abundance in early embryogenesis and generally decreasing towards late embryogenesis (with a second minor peak at Day11). Also in *Picea abies* somatic embryogenesis a *Histone 3* was found differentially expressed and down-regulated in the late embryogeny phase [60]. Histone H3 is a known phosphorylation target, in a cell cycle-dependent manner, of all three Arabidopsis Aurora kinases [61, 62]. Interestingly, a pine homolog of Aurora-2 (Pp32543) is present in the same cluster. Additionally, the H1 (linker) histones, putatively encoded by Pp46360, Pp14332 and Pp46359 pine transcripts following an overall profile presenting a peak of expression at D11/D15 stages (cluster 6 or 10), have been reported as involved in DNA methylation and demethylation, cell-cycle progression, and plant development (reviewed by [63]). A microarray analysis of somatic embryogenesis material from *Picea abies* revealed up-regulation of *Histone H1* in proembryogenic masses (PEMs) one day after withdrawal of plant growth regulators, when PEM-to-embryo transition is induced, and in the transition from proliferation to differentiation of early embryos [38].

Genes associated with chromatin formation or remodelling also appeared differentially expressed across pine embryo development. A *Serrated and early flowering*/ *SWR1 complex subunit 6* (*SEF/SWC6*) pine homolog (Pp30270) increased its expression throughout embryo development (cluster 7). *SEF* encodes a subunit of SWR1 chromatin-remodelling complex, which is responsible for the ATP-dependent replacement of histone H2A by H2A.Z variant, and is associated with flowering repression in Arabidopsis by means of positive regulation of the flowering repressors *FLOWERING LOCUS C* (*FLC*) and *MADS-AFFECTING FLOWERING 4* (*MAF4*) [64–66]. Two other SNF2-related chromatin remodelling putative transcripts follow different expression profiles. Pp14163, the putative pine homolog of *CHROMATIN REMODELING 31* (*CHR31*) peaked at Day5 and Day15, while the homolog of *RAD5* (Pp26994), was over-represented at early embryogenesis, pointing to a developmental stage dependent expression of these genes. Also peaking in early embryos was Pp34781, a putative *FASCIATA 1* (*FAS1*) pine homolog encoding one of the three subunits of the histone chaperone Chromatin Assembly Factor-1 (CAF-1). Its Arabidopsis counterpart is required during post-embryonic development, for proper organization and function of both apical meristems, however it appears not to be needed during embryo development, at least during Arabidopsis late embryogenesis [67].

The transcriptome of *P. pinaster* embryogenesis is abundant in transcripts possibly encoding enzymes for post-translational modification of histone subunits, in particular E2 ubiquitin-conjugating enzymes. Pp30887, a putative pine homolog of E2 ubiquitin-conjugating enzyme 28 gene, is differentially expressed and up-regulated in early and middle embryogenesis (cluster 1). These proteins are known to perform H2B monoubiquitination that has been associated with transcriptional activation (reviewed in [68]). Histone lysine methylation also plays a relevant role in pine embryogenesis considering the identification of the differentially expressed transcripts Pp44003 and Pp33894, putatively coding for an ARABIDOPSIS TRITHORAX-RELATED PROTEIN 6 (ATXR6) (in cluster 3) and a SU(VAR)3–9-RELATED protein 5 (SUVR5) (in cluster 10), respectively. ATXR5 and ATXR6, involved in the repressive chromatin modification H3K27me1, contribute to keep the constitutive heterochromatin status, in most cases of transposons and other repetitive and silent elements, and to prevent re-replication to occur in the same cell cycle [69, 70]. On the other hand, SUVR5 is responsible for the repressive chromatin modification H3K9me2, independently of the presence of DNA methylation [71], whose presence is usually associated to transposon silencing and DNA methylation control, being predominant in pericentromeric/centromeric regions [72].

A putative homolog of VARIANT IN METHYLATION 1/ORTHRUS 2 (VIM1/ORTH2) gene, a methylcytosine-binding protein that collaborates with DNA METHYLTRANSFERASE 1 (MET1) to promote CpG methylation and centromeres organization [73, 74], was also found in our data (Pp11214) showing a maximum expression in the earlier embryo stages (cluster 3) and decreasing towards the mature embryo. In *Picea abies*, *VIM1* was found differentially expressed between somatic embryos in the early morphogenesis stage grown under two distinct temperature conditions (18 °C and 30 °C), being up-regulated at higher temperature [23]. However, Pp3794, which is an homolog of the plant *DOMAINS REARRANGED METHYLTRANSFERASE 2* (*DRM2*), a major de novo DNA methyltransferase gene responsible for DNA methylation in all sequence contexts (CG, CHG and CHH) (reviewed by [75]), followed an expression profile peaking at Day5, but increasing from middle embryogenesis towards maturation (cluster 5). DRM2 maintains CHH methylation through de novo methylation, typically within the RNA-directed DNA methylation (RdDM) pathway (reviewed in [76]). Although the expression of the pine putative DRM2 peaks before reaching maturation, a tendency for a steady increase from the middle embryo stages up to the mature embryo was detected. This observation is in agreement with studies in Arabidopsis showing that mature embryos exhibit saturation of the CHH methylation sites, and a higher activity of RdDM and expression of *DRM2* when comparing with early embryos [77, 78].

Finally, many transcripts associated with RNA silencing have been found in the pine embryo transcriptome. Within this group, it is worth highlighting Pp12441, a putative pine homolog of the RNA silencing player ARGONAUTE 2 which has been associated with biotic stress response [79], up-regulated during early and middle embryogenesis (cluster 1).

## Conclusions

This work provides an additional resource to help understand the gene regulation and major events associated with embryogenesis progression in conifers. By using RNA-seq technology to access the genes being expressed at specific embryo developmental stages, we have extended the previously published transcriptome profiling of maritime pine zygotic embryogenesis which had been obtained with DNA microarray hybridization technology. One such important outcome is that carbohydrate transport and metabolism was found clearly over-represented in early embryo stages. Either the analysis of functional categories assigned to eggNOGs, or the enrichment analysis of the differentially expressed transcripts and identification of many putative glycoside hydrolases and carbohydrate transport genes, point towards their relevant role in pine embryo development. Another relevant outcome providing strong support to previous studies is the predominance, during early and middle embryogenesis, of several events of chromatin remodelling evidenced by an active synthesis of histones and their post-translational modifiers associated to increased transcription, as well as silencing of transposons.

While there is no genome published for *P. pinaster,* this transcriptome of reference for pine zygotic embryogenesis is useful to the plant research community focused on the improvement of the vegetative propagation of conifers through somatic embryogenesis.

## Methods

### Plant material

Immature female cones were collected from open-pollinated *P. pinaster* Ait. trees growing in a clonal seed orchard at Mata Nacional do Escaroupim, Portugal (Longitude 8°44’W, latitude 39°4’N). This seed orchard was established by top grafting of clones genetically selected in a half-sib progeny test. The plus trees were originally selected in Mata Nacional de Leiria (Portugal) in 1963/64 [80]. The trees are part of an experimental plantation established for research on land of the Portuguese government. The cones were obtained from INIAV (Oeiras, Portugal), *Ministério da Agricultura, Florestas e Desenvolvimento Rural*, and were provided upon permission by the forest engineers Alexandre Aguiar and Isabel Carrasquinho, complying with institutional and national guidelines.

The collection period occurred between mid June and end of July. Seeds were removed and used to isolate embryos as previously described in [36]. Each embryo was quickly evaluated for developmental stage following the staging system described by [30], the suspensor was removed, and the embryo immediately frozen in liquid nitrogen into different pools according to the stage. Five different embryo developmental stages were considered as follows: Day0 included the early embryo stages T0, T1 and T2; Day5 included the pre-cotyledonary embryo stages T3 and T4; Day11 included the early cotyledonary embryo stage T4B; Day15 included the cotyledonary embryo stage T5; and Day25 included the mature embryo stage T7. Depending on the embryo stages, each pool contained 20–65 zygotic embryos. Several separate pools were prepared for each stage and samples were stored at − 80 °C until further analysis.

### RNA extraction and sequencing

RNA extraction from each embryo pool was performed with RNeasy Plant Mini kit (Qiagen, Valencia CA, USA), using buffer RLC, according to the manufacturer’s instructions. RNA yield and purity were determined using ND-1000 spectrophotometer (NanoDrop, Wilmington DE, USA), and integrity was checked by electrophoresis in 0.8% agarose gel and staining with RedSafe™ Nucleic Acid Staining Solution (iNtRON Biotechnology). RNA samples were cleaned from DNA contamination using RNase-Free DNase I (Qiagen), according to manufacturer’s instructions. Total RNA samples from the five embryo stage pools (one biological replicate), were sent to the sequencing service provider where Illumina RNA-seq libraries were prepared and sequenced using the HiSeq 2000 platform.

### RNA-seq data pre-processing and comprehensive assembly

A comprehensive transcriptome assembly approach [31] reviewed in [32] was adopted to generate the reference transcriptome (Software was run with default parameters unless otherwise indicated): The raw reads were filtered with Trimmomatic (v 0.32; [81]) using the default options for paired-end (PE) reads to remove *Illumina* adaptor sequences, reads with low quality or complexity (SLIDINGWINDOW:4:5), 5 bp from both ends (LEADING:5 TRAILING:5) and reads shorter than 25 bp (MINLEN:25). All the clean reads were used for de novo assembly with Trinity, but only pairs where both reads remained were used for the guided assembly or expression analysis. Trinity (v 2.0.6; [33]) was used to generate a de novo assembly with default parameters, plus “--min_glue 4 --CuffFly --group_pairs_distance 600 --genome_guided_max_intron 10000”. The *P. taeda* genome and annotations (v 1.01) were downloaded from the genome project at the University of California [34]. Clean pairs of reads from each embryo stage were independently aligned to this *P. taeda* genome using GSNAP without gene annotation (v 2014-08-04; [82]). We only used “concordant paired” alignments where both reads in a pair align with a minimum length of 25 bp (50 bp per alignment), in the right forward-reverse orientation and insert length distance. The read alignments from all the stages, together with the *P. taeda* gene annotation, were used as input in Cufflinks (v. 2.2.1; [83]) to reconstruct another set of transcripts. The final step involves combining both set of transcripts, from Trinity and Cufflinks: These transcripts were aligned to the *P. taeda* genome with GMAP (v 2014-12-22, [84]), and the aligning transcripts were clustered by locus with PASA (release 20,140,417; [85]). However, the transcripts that did not align on the *P. taeda* genome were clustered by sequence using CD-HIT with “-pid 1” (v 4.6.3; [86]), later validated by checking the presence of a clear ORF within each of them with TransDecoder (v 2.0.1; [87]) using all the *Viridiplantae* proteins in UniProt as reference, and finally concatenated to the previous transcripts (those clustered by locus). To produce the final comprehensive transcriptome, we filtered out the 602 short transcripts without an ORF and shorter than 200 bp. These 602 transcripts are listed in Additional file 2.

### Functional annotation

Transcripts were compared with the NCBI non-redundant (nr) and Arabidopsis TAIR protein databases using NCBI BLASTX with an E-value of 1e-10. Results were imported in Blast2GO [88] to annotate the GO terms, enzymatic protein codes and KEGG pathways. The conserved motifs and structures in the transcripts were identified by comparison against the motifs databases in EBI InterPro (http://www.ebi.ac.uk/interpro/interproscan.html). We used the Plant Transcription Factor database (PLNTFDB, http://plntfdb.bio.uni-potsdam.de/) as reference to identify the TFs and other transcriptional regulators in our transcriptome. The database contains close to 30,000 protein sequences of experimentally-identified elements from diverse plant species, and their classification in families according to their protein domains by HMM methods. The sequences of the differentially expressed transcripts were aligned to the PLNTFDB using BLASTX and a minimum E-value of 1e-10. We considered any transcript with a result under that threshold as a TF/transcriptional regulator, and annotated it within the family of the homologous with a lower E-value.

All ORF possibilities were generated from the newly assembled transcriptome using TransDecoder as previously described, but only the longest ORF per transcript was retained in the final *P. pinaster* proteome. We classified any assembled transcript where TransDecoder could not identify an ORF as non-coding. The transcriptomes of other conifers were downloaded from the PineRefSeq project at University of Davis. The proteome for each of them was built in a similar way as for *P. pinaster* with TransDecoder. Best reciprocal hits (BRH) were identified by aligning all the proteins against each other with BLASTP with an E-value of 1e-5. Any annotation from the close relatives was assigned back to the original *P. pinaster* transcript. The proteins from *P. pinaster, P. taeda* and *P. lambertiana* were clustered together according the eggNOG group of their respective best orthologous sequenced in EBI’s eggNOG database of functionally annotated proteins. Each protein was firstly aligned to a database of proteins that have already been categorized, and then annotated with the eggNOG number of its best homolog. Clusters of proteins were made among the three conifer species by concatenating the proteins annotated with the same eggNOG number. The annotated database of protein sequences and descriptions is available to download at EMBL (http://eggnogdb.embl.de/).

### Analysis of expression, gene enrichment and clustering

The clean reads from each of the developing stages were aligned to the comprehensive transcriptome with Bowtie (v 2.2.5; [89]) and retaining only pairs of reads mapping in the right distance and orientation (−-very-sensitive -X 1000 --no-mixed --no-discordant). From these alignments, the abundance of each transcript was quantified in each stage using eXpress (v. 1.5.1; [90]) to produce a table of counts that was used downstream in edgeR [91]. Transcripts with less than 10 counts were discarded at this point. Due to the technical difficulties in isolating enough amounts of zygotic embryos at the very early stages of development, we do not have replicated libraries. To minimize the impact of the lack of replicates, we followed the protocol recommended by the developers in such cases [92]: a common dispersion was calculated for the whole dataset with “x = *DGEList(round(read.delim(‘eXpress.eff_counts’)),group=c(1,1,1,1,1); x = calcNormFactors(x); x = estimateDisp(x);*”, and later differentially expressed transcripts were identified between each pair of consecutive stages at FDR < 0.05 using edgeR’s exact test “exactTest()”, which allows both common dispersion and single factor experiments (time in ours).

Differentially expressed transcripts were divided in clusters according to the normalized number of aligned reads in each stage by K-means clustering implemented in Mayday [93] based on Euclidian correlation between expression values. The list of transcripts in each cluster was used in Blast2GO to identify the enriched GO terms. The enrichment analysis was based on a F-fisher test (FDR < 0.05). The relation among GO terms was assigned using REVIGO with the Resvik algorithm option [94] and plotted in R with the Treemap library (github.com/mtennekes/treemap.git). To build the expression heatmap by functional categories, the counts of each transcript belonging to a protein group in such category were added up, and later transformed in Z-scores, clustered, and plotted in a heatmap using Mayday [93].

### Expression validation by RT-qPCR

A subset of genes from the de novo assembled *P. pinaster* transcriptome was selected, based on differential expression and/or epigenetic-related annotation, to validate the RNA-seq results by RT-qPCR. Primer3Plus (http://primer3plus.com/cgi-bin/dev/primer3plus.cgi) was used to design the RT-qPCR primers, with the following conditions: 50–60% of GC content and Tm between 50 and 65 °C (according to Nearest Neighbor Tm); should bind the 5′ or 3′ less conserved regions of the transcript; the amplicon size of 75–200 bp (Additional file 18). The quality of the primers was verified with PCR Primer Stats (http://www.bioinformatics.org/sms2/pcr_primer_stats.html) and OligoAnalyzer 3.1 (https://eu.idtdna.com/calc/analyzer).

RNA samples were first quantified in Qubit 3.0 Fluorimeter using the RNA BR Assay kit (Thermo Fisher Scientific). The cDNA synthesis was performed using the Transcriptor High Fidelity cDNA Synthesis Kit (Roche Diagnostics), according to the manufacturer’s instructions in the Standard Procedure for Quantitative RT-PCR, adding 1000 ng of total RNA per 20 uL reaction mix. Three biological replicates were prepared for each embryo stage pool.

All qPCR experiments were performed in a LightCycler 480 (Roche Diagnostics) with 96-well white plates (Roche Diagnostics), where each 20 uL qPCR reaction mixture included 1X SYBR Green I Master (Roche Diagnostics), 500 nM of each primer and 2 uL of 1:20 diluted cDNA. Three technical replicates were prepared for each biological replicate. The amplification program was the same for all genes, with slight differences in the annealing temperatures: 95 °C for 10 min, 40 cycles of 10 s at 95 °C, 20 s at annealing temperature and 8 s at 72 °C (annealing temperatures in Additional file 18). To check for the presence of a single peak upon amplification, melting curves were obtained. In order to discard any possibility of remnant gDNA contamination, control samples were prepared from the same RNAs but in the absence of the Reverse Transcriptase enzyme, and used as templates in qPCR amplification with the ELF1A primers, where the absence of signal proved the effectiveness of the previous RNase-free DNase I (Qiagen) treatment. As additional controls, non-template controls (NTC) and positive controls were included in all runs. Additionally, three biological replicates of a pool sample, prepared from equal amounts of total RNA from each of the five embryo stage pools, were also included in all runs. The efficiency of each pair of primers was manually calculated according to [95], using the Cp values retrieved by the ROCHE LC480 software (Additional file 18). The relative expression of each gene of interest (GOI) was efficiency corrected as described in [95], using ELF1A, ATUB and Histo3 as reference genes, which showed up as reliable reference genes in a previous study on *P. pinaster* somatic embryogenesis [96].

The data obtained from the RNA-seq experiment and the RT-qPCR were compared. From the RNA-seq a logarithmic ratio of base 2 between the counts (from eXpress) of a gene in each developmental stage and the mean counts of the same gene in all developmental stages were made. A similar approach was followed for the data obtained by RT-qPCR by doing a logarithmic ratio of base 2 between the normalized quantities (delta-delta-Ct) of the gene of interest in each developmental stage and the mean normalized quantities of the same gene in all developmental stages in analysis. If expression could not be detected by RT-qPCR after 35 cycles, we assigned an expression value lower than the previous detected.

## Additional files

Additional file 1: List of all sequences of proteins in the final proteome encoded by the reference transcriptome of *P. pinaster* zygotic embryogenesis. Only the longest ORF possibility per transcript was translated into protein sequence to generate the final proteome. (FASTA 14164 kb)

Additional file 2: Reference transcriptome of *P. pinaster* zygotic embryogenesis with annotations from BRH to the proteomes of *P. taeda*, *P. lambertiana* and *A. thaliana*, and from homology to proteins in NCBI databases. Table S1: List of assembled transcripts, longest ORFs, and corresponding predicted proteins. Table S2: Annotation information for the predicted proteins derived from the longest ORFs. Table S3: List of short non-coding transcripts without an ORF that were excluded. (XLSX 11164 kb)

Additional file 3: E-value distribution of the BLAST hits resulting from the BLASTX alignment of the *P. pinaster* transcriptome to the NCBI non-redundant proteins database. (PNG 74 kb)

Additional file 4: The functional annotation of *P. pinaster* transcriptome done with Blast2GO generated different charts. The “data distribution” chart shows the distribution of un-blasted (with BLAST (without hits)), blasted (with BLAST hits), mapped (with mapping) and annotated (with GO annotation) transcripts over the whole transcriptome. The “GO mapping distribution” chart is a representation of the amount of GO terms assigned to each sequence during the GO Mapping step. The third chart represents the number of annotations achieved at distinct GO levels (0-to-15), listing the GO terms by biological process (P), molecular function (F) and cellular component (C). The “annotation distribution” chart shows the number of sequences annotated with different amounts of GO terms. (PDF 2905 kb)

Additional file 5: Distribution of the sequence similarities (percentage) that were calculated for the BLAST hits. (PNG 79 kb)

Additional file 6: Distribution of species to which most transcripts were aligned when only considering the Top-BLAST hits. (PNG 103 kb)

Additional file 7: InterProScan (IPS) results showing the number of transcripts with and without IPS as well as with GO terms retrieved by this annotation step. (PNG 45 kb)

Additional file 8: Distribution of the number of GO terms, retrieved by the Blast2GO mapping step, per database resource. (PNG 24 kb)

Additional file 9: List of proteins from *P. pinaster*, *P. taeda* and *P. lambertiana* clustered together according to the eggNOG group of their respective best orthologous sequenced in EMBL’s eggNOG database of functionally annotated proteins. (TXT 3127 kb)

Additional file 10: Number of proteins from *P. pinaster*, *P. taeda* and *P. lambertiana* clustered together according to the eggNOG group of their respective best orthologous sequenced in EMBL’s eggNOG database of functionally annotated proteins. Information on each eggNOG group is complemented by a general category and its description. (XLSX 844 kb)

Additional file 11: Transcripts abundance per developmental time point (0D_eff_counts to 25D_eff_counts). (XLSX 4407 kb)

Additional file 12: Results from the edgeR analysis for the identification of differentially expressed transcripts between each pair of consecutive stages at FDR < 0.05. Fold-change (FC), counts per million (CPM) and *p*-value data are shown per developmental transition and transcript. Table S4: Complete list of transcripts analysed with edgeR. Table S5: Exclusive list of 1738 differentially expressed transcripts along embryo development, including the number of the cluster of expression profile. (XLSX 2943 kb)

Additional file 13: REVIGO TreeMap representation of GO terms enrichment analysis associated with biological process GO terms found in the list of 204 differentially expressed transcripts down-regulated in transition from Day0 to Day5. (PNG 62 kb)

Additional file 14: REVIGO TreeMap representation of GO terms enrichment analysis associated with biological process GO terms found in the list of 594 differentially expressed transcripts up-regulated in transition from Day0 to Day5. (PNG 50 kb)

Additional file 15: REVIGO TreeMap representation of GO terms enrichment analysis associated with biological process GO terms found in the list of 344 differentially expressed transcripts down-regulated in transition from Day15 to Day25. (PNG 167 kb)

Additional file 16: REVIGO TreeMap representation of GO terms enrichment analysis associated with biological process GO terms found in the list of differentially expressed transcripts included in cluster 2. (PNG 53 kb)

Additional file 17: REVIGO TreeMap representation of GO terms enrichment analysis associated with biological process GO terms found in the list of differentially expressed transcripts included in cluster 3. (PNG 75 kb)

Additional file 18: Transcripts with BRHs validated by relative RT-qPCR. The order of preference for annotating each *P. pinaster* transcript after its homologs is: *A. thaliana*, *P. taeda*, and *P. lambertiana*. (DOCX 15 kb)

## Abbreviations

BRH: Best reciprocal hits
GO: Gene ontology
IPS: InterPro database
NGS: Next-generation sequencing
ORF: Open reading frame
RNA-seq: RNA sequencing
