# Supplementary figures and images for: Comprehensive assembly and analysis of the transcriptome of maritime pine developing embryos
Source: BMC Plant Biol. 2018 Dec 29;18:379. doi: 10.1186/s12870-018-1564-2 (PMC6310951; doi:10.1186/s12870-018-1564-2)

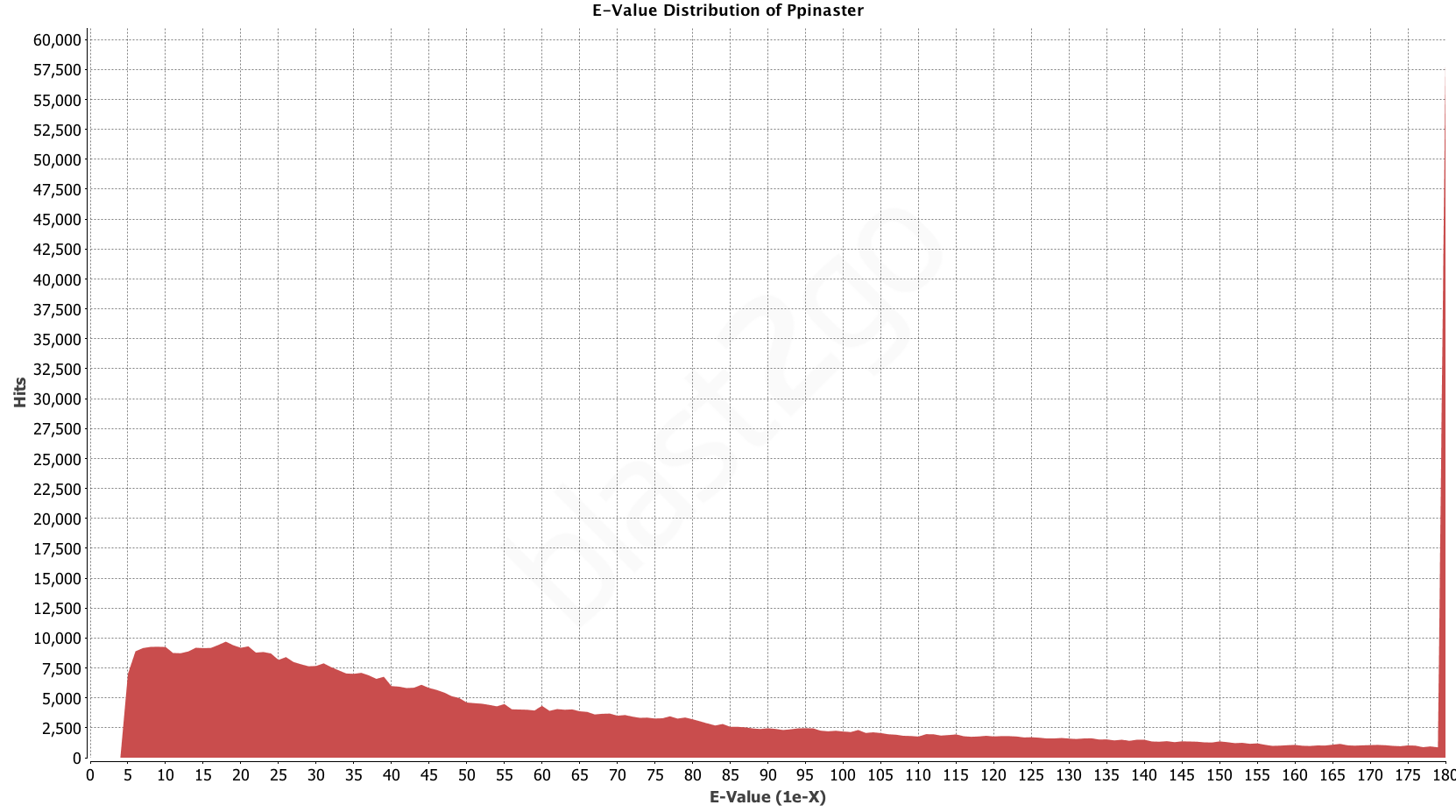

Supplement: Supplementary file 3 — E-value distribution of the BLAST hits resulting from the BLASTX alignment of the P. pinaster transcriptome to the NCBI non-redundant proteins database. (PNG 74 kb) [file 12870_2018_1564_MOESM3_ESM.png]

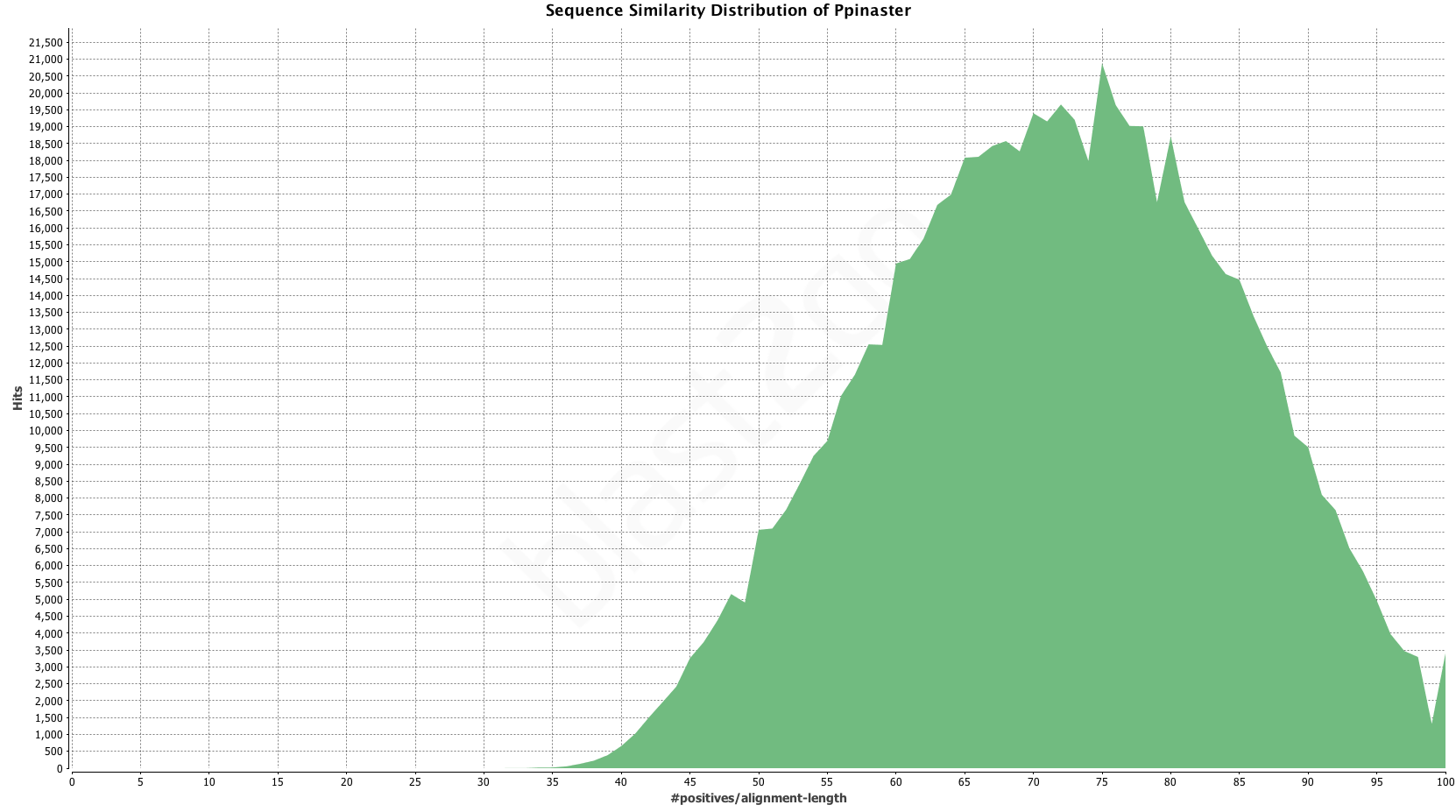

Supplement: Supplementary file 5 — Distribution of the sequence similarities (percentage) that were calculated for the BLAST hits. (PNG 79 kb) [file 12870_2018_1564_MOESM5_ESM.png]

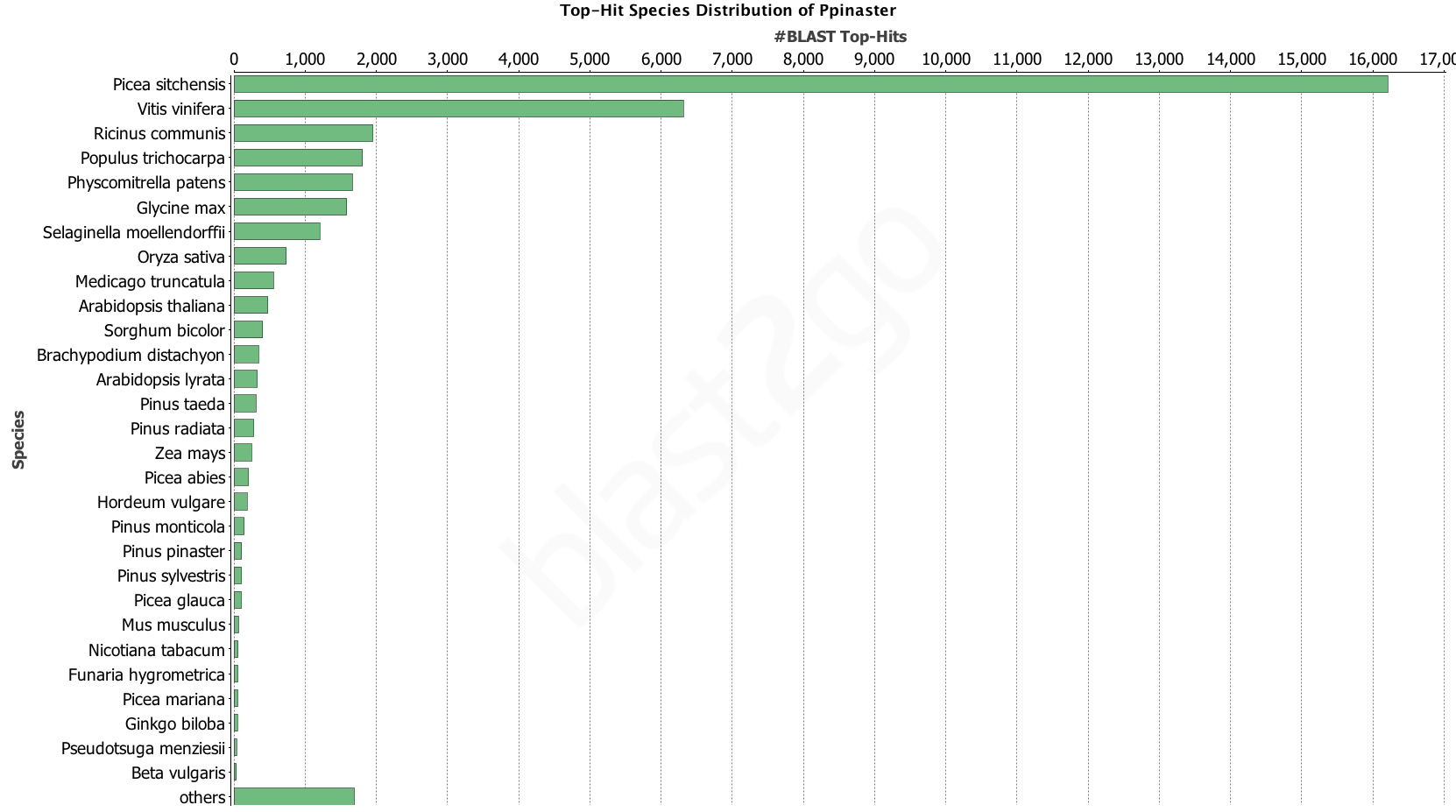

Supplement: Supplementary file 6 — Distribution of species to which most transcripts were aligned when only considering the Top-BLAST hits. (PNG 103 kb) [file 12870_2018_1564_MOESM6_ESM.png]

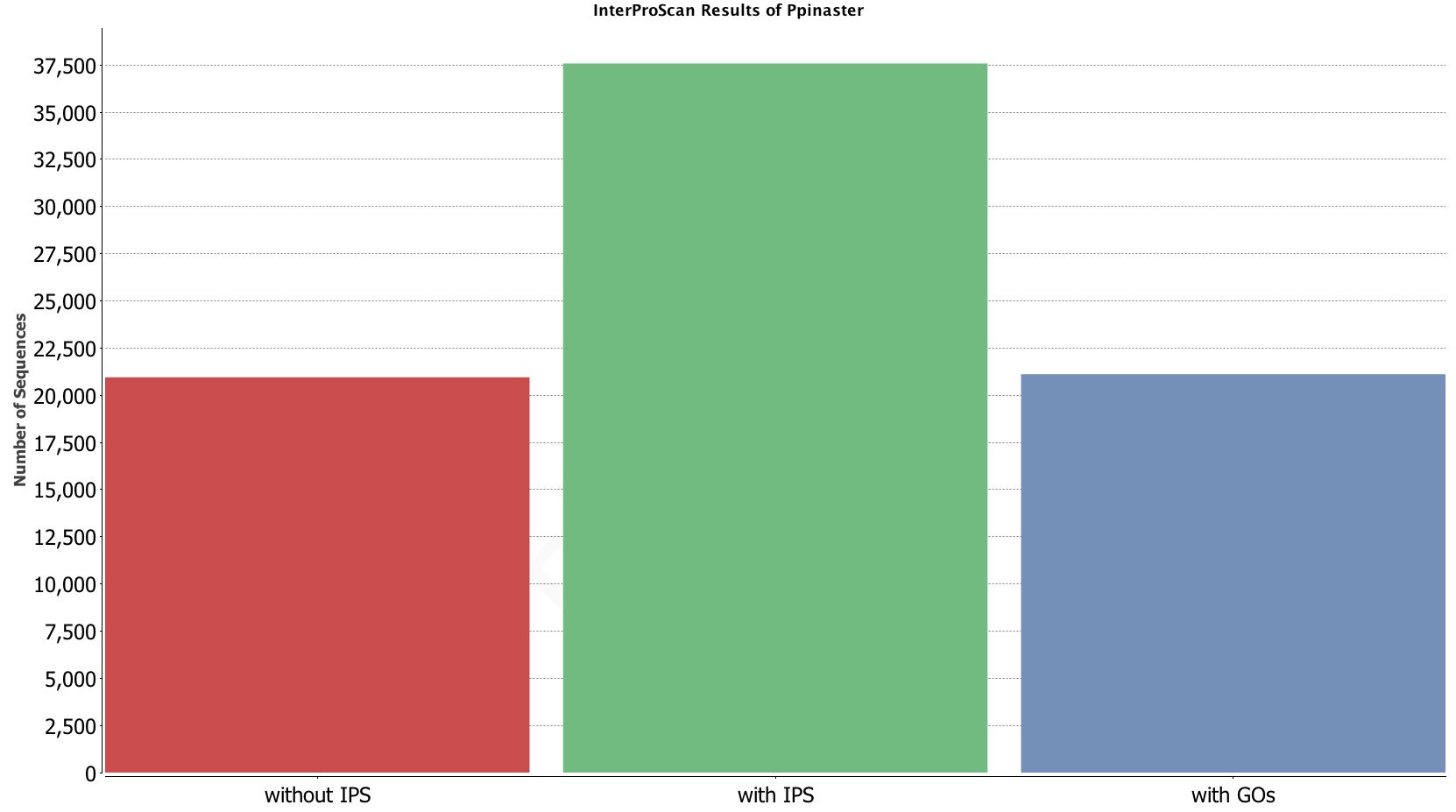

Supplement: Supplementary file 7 — InterProScan (IPS) results showing the number of transcripts with and without IPS as well as with GO terms retrieved by this annotation step. (PNG 45 kb) [file 12870_2018_1564_MOESM7_ESM.png]

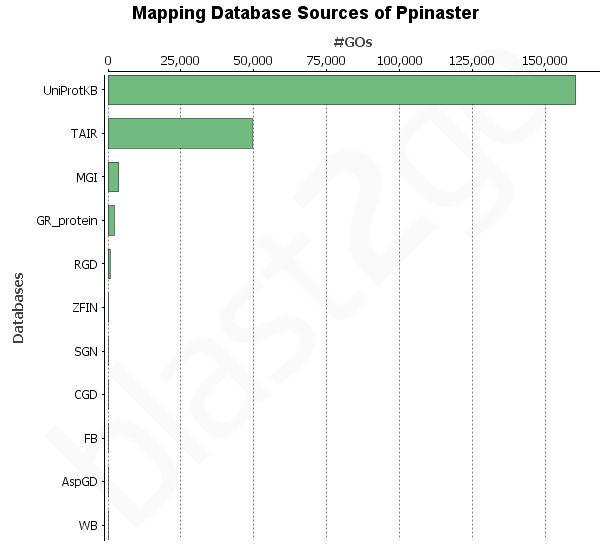

Supplement: Supplementary file 8 — Distribution of the number of GO terms, retrieved by the Blast2GO mapping step, per database resource. (PNG 24 kb) [file 12870_2018_1564_MOESM8_ESM.png]

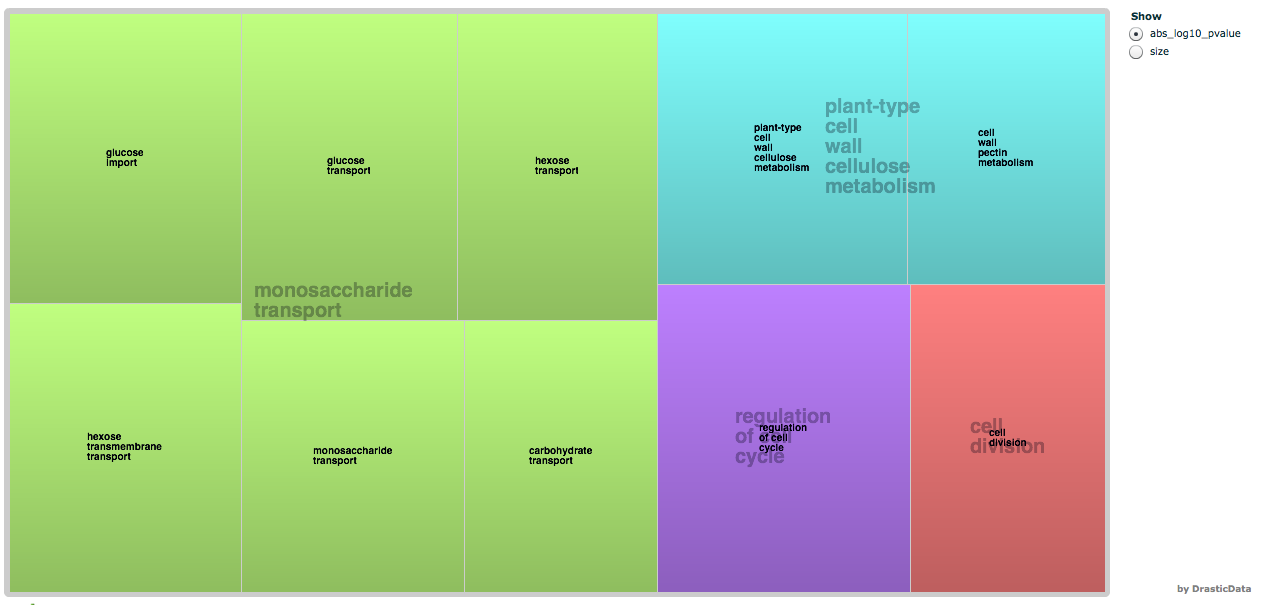

Supplement: Supplementary file 13 — REVIGO TreeMap representation of GO terms enrichment analysis associated with biological process GO terms found in the list of 204 differentially expressed transcripts down-regulated in transition from Day0 to Day5. (PNG 62 kb) [file 12870_2018_1564_MOESM13_ESM.png]

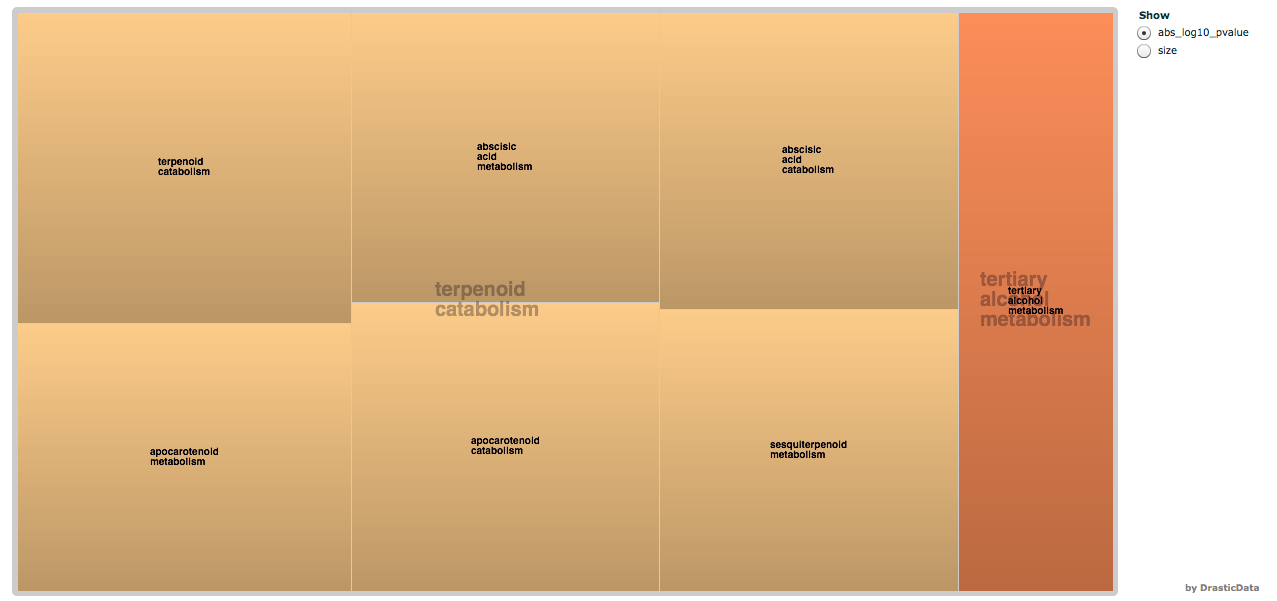

Supplement: Supplementary file 14 — REVIGO TreeMap representation of GO terms enrichment analysis associated with biological process GO terms found in the list of 594 differentially expressed transcripts up-regulated in transition from Day0 to Day5. (PNG 50 kb) [file 12870_2018_1564_MOESM14_ESM.png]

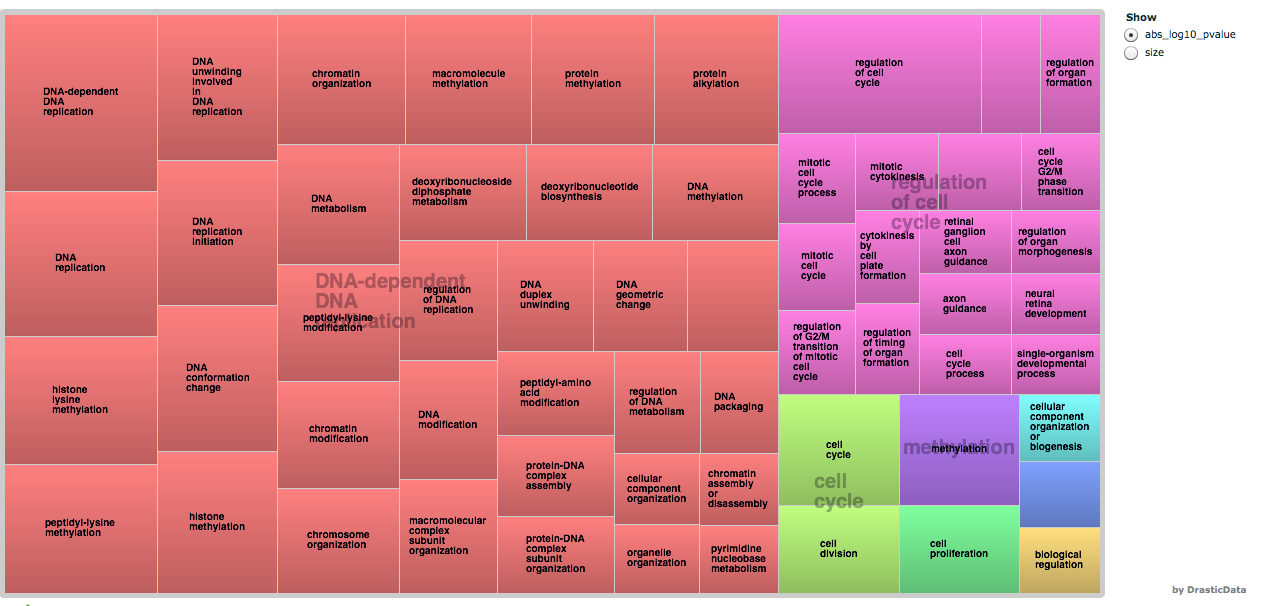

Supplement: Supplementary file 15 — REVIGO TreeMap representation of GO terms enrichment analysis associated with biological process GO terms found in the list of 344 differentially expressed transcripts down-regulated in transition from Day15 to Day25. (PNG 167 kb) [file 12870_2018_1564_MOESM15_ESM.png]

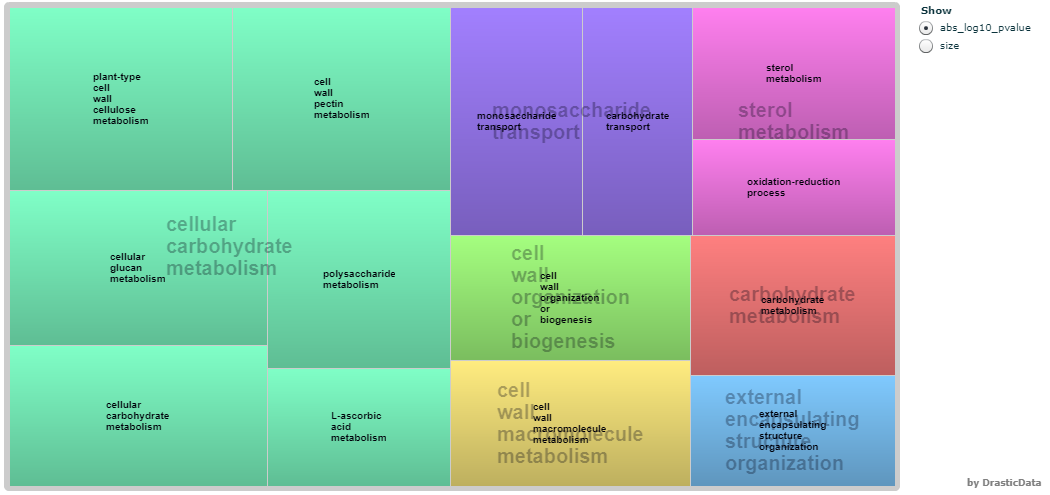

Supplement: Supplementary file 16 — REVIGO TreeMap representation of GO terms enrichment analysis associated with biological process GO terms found in the list of differentially expressed transcripts included in cluster 2. (PNG 53 kb) [file 12870_2018_1564_MOESM16_ESM.png]

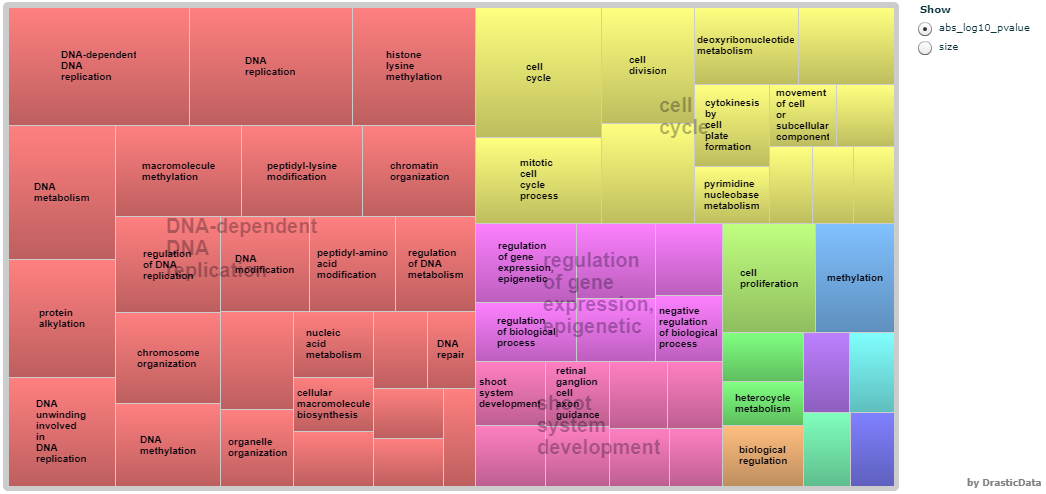

Supplement: Supplementary file 17 — REVIGO TreeMap representation of GO terms enrichment analysis associated with biological process GO terms found in the list of differentially expressed transcripts included in cluster 3. (PNG 75 kb) [file 12870_2018_1564_MOESM17_ESM.png]
