# Supplementary material for: Comprehensive assembly and analysis of the transcriptome of maritime pine developing embryos
Source: BMC Plant Biol. 2018 Dec 29;18:379. doi: 10.1186/s12870-018-1564-2 (PMC6310951; doi:10.1186/s12870-018-1564-2)
Supplement: Supplementary file 4 — The functional annotation of P. pinaster transcriptome done with Blast2GO generated different charts. The “data distribution” chart shows the distribution of un-blasted (with BLAST (without hits)), blasted (with BLAST hits), mapped (with mapping) and annotated (with GO annotation) transcripts over the whole transcriptome. The “GO mapping distribution” chart is a representation of the amount of GO terms assigned to each sequence during the GO Mapping step. The third chart represents the number of annotations achieved at distinct GO levels (0-to-15), listing the GO terms by biological process (P), molecular function (F) and cellular component (C). The “annotation distribution” chart shows the number of sequences annotated with different amounts of GO terms. (PDF 2905 kb) [file 12870_2018_1564_MOESM4_ESM.pdf]

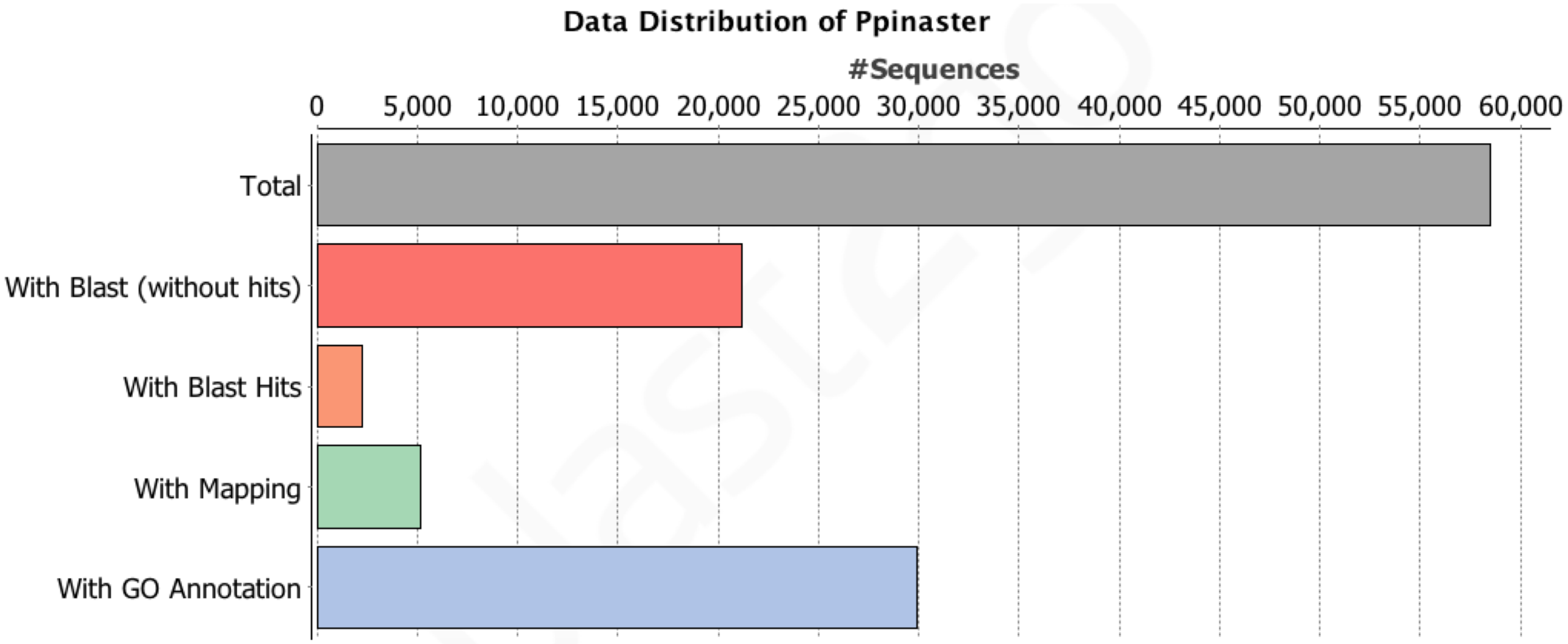
Data Distribution of Ppinaster
#Sequences
0
5,000
10,000
15,000
20,000
25,000
30,000
35,000
40,000
45,000
50,000
55,000
60,000
Total
With Blast (without hits)
With Blast Hits
With Mapping
With GO Annotation

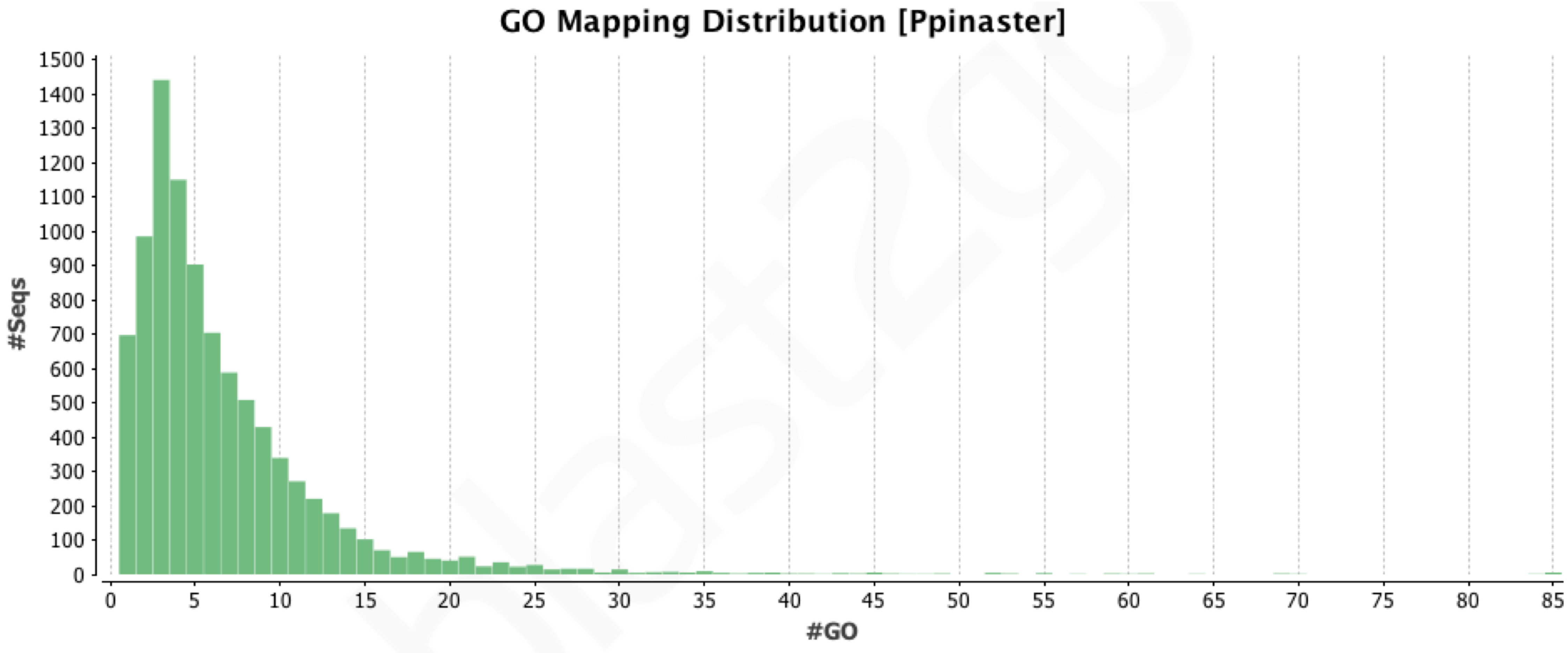
GO Mapping Distribution [Ppinaster]
#Seqs
0
100
200
300
400
500
600
700
800
900
1000
1100
1200
1300
1400
1500
#GO
0
5
10
15
20
25
30
35
40
45
50
55
60
65
70
75
80
85

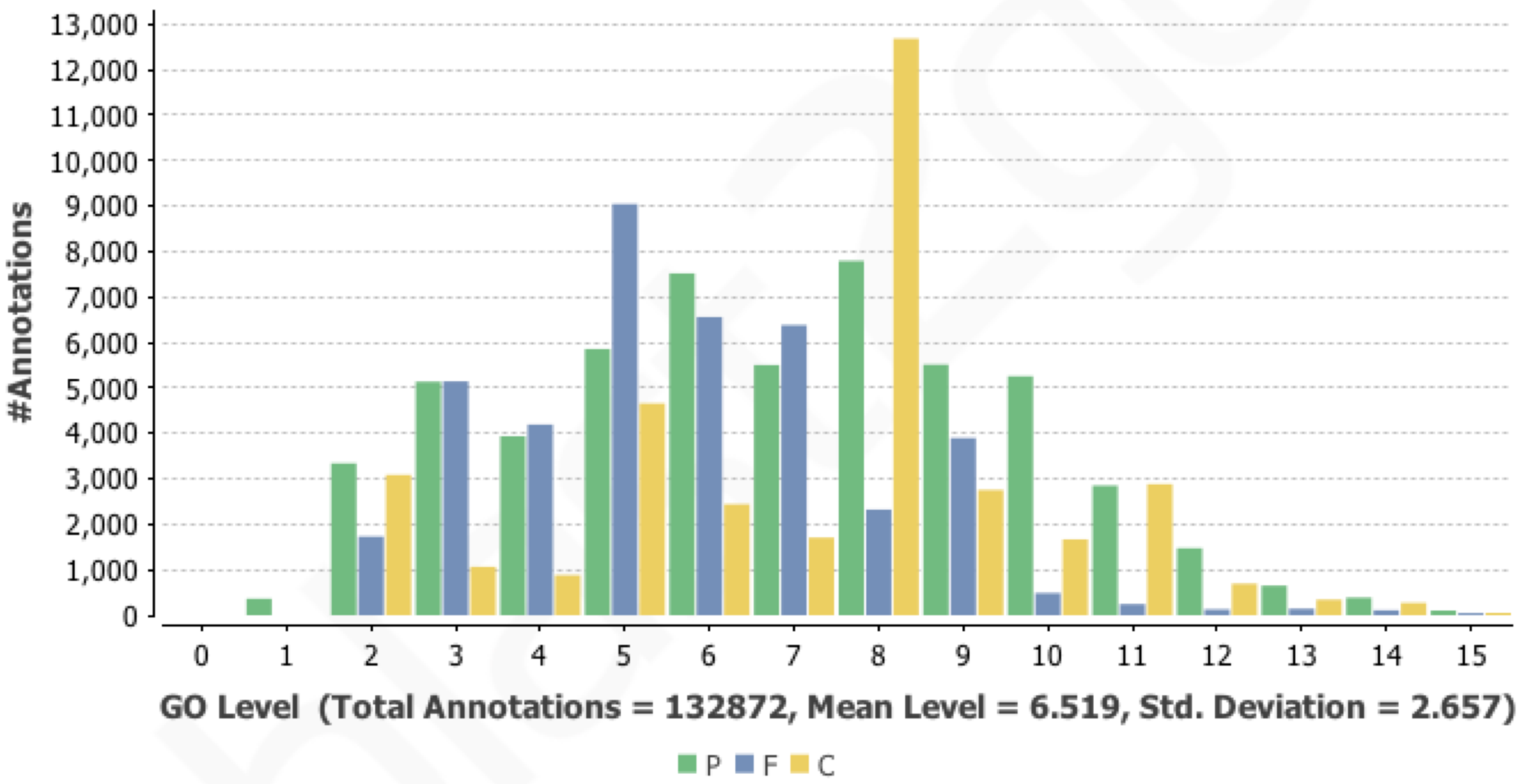
#Annotations
13,000
12,000
11,000
10,000
9,000
8,000
7,000
6,000
5,000
4,000
3,000
2,000
1,000
0
0 1 2 3 4 5 6 7 8 9 10 11 12 13 14 15
GO Level (Total Annotations = 132872, Mean Level = 6.519, Std. Deviation = 2.657)
P F C

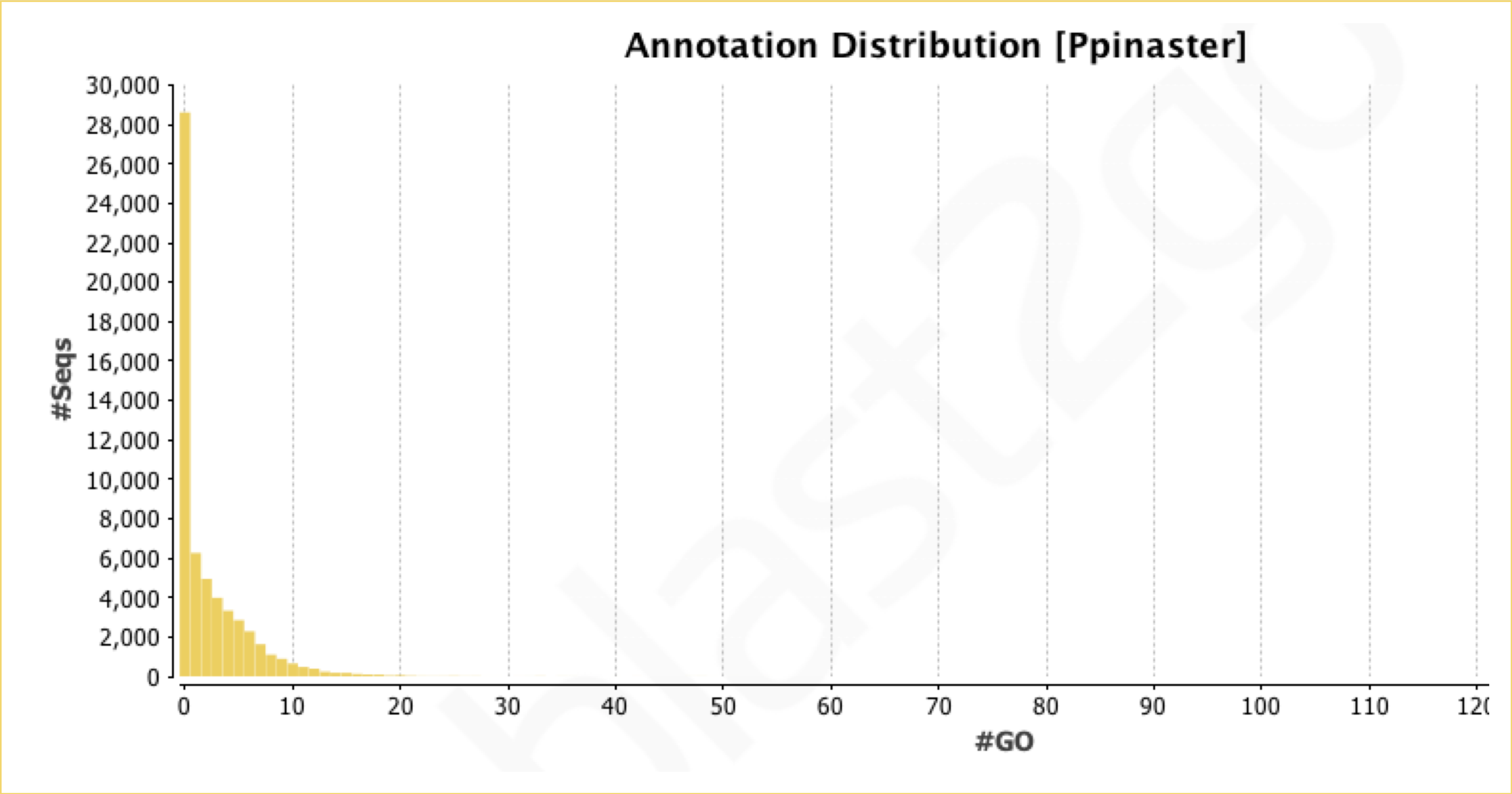
Annotation Distribution [Ppinaster]
#Seqs
30,000
28,000
26,000
24,000
22,000
20,000
18,000
16,000
14,000
12,000
10,000
8,000
6,000
4,000
2,000
0
0
10
20
30
40
50
60
70
80
90
100
110
#GO
