# Supplementary material for: Comprehensive assembly and analysis of the transcriptome of maritime pine developing embryos
Source: BMC Plant Biol. 2018 Dec 29;18:379. doi: 10.1186/s12870-018-1564-2 (PMC6310951; doi:10.1186/s12870-018-1564-2)
Supplement: Supplementary file 18 — Transcripts with BRHs validated by relative RT-qPCR. The order of preference for annotating each P. pinaster transcript after its homologs is: A. thaliana, P. taeda, and P. lambertiana. (DOCX 15 kb) [file 12870_2018_1564_MOESM18_ESM.docx]

| Cluster | Transcript ID | Annotation | Primer sequence (forward/reverse) | Primers efficiency Tannealing Amplicon size |
| --- | --- | --- | --- | --- |
| K5 | Pp3794 | PREDICTED: DNA (cytosine-5)-methyltransferase DRM2-like | GCAAGACCTGAAATCGTCCATC/ TGGCACCGAAAGATGTTTGG | 1.963 56ºC 147 bp |
| N/A | Pp29536 | PREDICTED: DNA (cytosine-5)-methyltransferase 1-like | GGCTGGGTCACACTAATTCAAAG/ TGCTTCAGATCCACACTCCTG | 2.009 56ºC 105 bp |
| K2 | Pp34009 | PREDICTED: L-ascorbate oxidase homolog | AAACCATTGTCGGAGGAAGC/ AACCCATTGCGTTCTTGGTG | 2.093 60ºC 101 bp |
| N/A | Pp34388 | DEFECTIVE IN MERISTEM SILENCING 2, DMS2, DRD2, NRPD2, NRPD2A, NRPE2, NUCLEAR RNA POLYMERASE D2A, OCP1, OVEREXPRESSOR OF CATIONIC PEROXIDASE 1 | TTCGTTCCACTGTCTTCTCC/ TTCCTTACTTCCACGCCAAC | 1.987 56ºC 165 bp |
| N/A | Pp34678 | PREDICTED: transcriptional activator DEMETER-like | AACGTCCAGCAAGATCAGTG/ ATCCAAATCGATCCGCACAC | 1.889 60ºC 100 bp |
| K3 | Pp34781 | FAS1, FASCIATA 1, FUGU 2, FUGU2, NFB2, NUCLEOSOME/CHROMATIN ASSEMBLY FACTOR GROUP B | AACTTTGTTGCCGACTTCCG/ TGGCAGCAGGATTGTTGTTG | 1.944 56ºC 103 bp |
| K2 | Pp38781 | ATGH9B13, GH9B13, GLYCOSYL HYDROLASE 9B13 | AGACAAGGAGGATGGATGGAAC/ TCAGGAGATAGGCTGGATTTC | 1.916 56ºC 135 bp |
| K10 | Pp46359 | histone H1.2 | CCCGCTTTTGATTTTGTCTGC/ CCTCGGCTTCTTTTCCTTTGC | 1.959 56ºC 159 bp |
